# *Cauliflower mosaic virus* protein P6 is a multivalent node for RNA granule proteins and interferes with stress granule responses during plant infection

**DOI:** 10.1093/plcell/koad101

**Published:** 2023-04-12

**Authors:** Gesa Hoffmann, Silvia López-González, Amir Mahboubi, Johannes Hanson, Anders Hafrén

**Affiliations:** Department of Plant Biology, Uppsala BioCenter, Swedish University of Agricultural Sciences, 75007 Uppsala, Sweden; Linnean Center for Plant Biology, 75007 Uppsala, Sweden; Department of Plant Biology, Uppsala BioCenter, Swedish University of Agricultural Sciences, 75007 Uppsala, Sweden; Linnean Center for Plant Biology, 75007 Uppsala, Sweden; Department of Plant Physiology, Umeå Plant Science Centre, Umeå University, 90736 Umeå, Sweden; Department of Plant Physiology, Umeå Plant Science Centre, Umeå University, 90736 Umeå, Sweden; Department of Plant Biology, Uppsala BioCenter, Swedish University of Agricultural Sciences, 75007 Uppsala, Sweden; Linnean Center for Plant Biology, 75007 Uppsala, Sweden

## Abstract

Biomolecular condensation is a multipurpose cellular process that viruses use ubiquitously during their multiplication. *Cauliflower mosaic virus* replication complexes are condensates that differ from those of most viruses, as they are nonmembranous assemblies that consist of RNA and protein, mainly the viral protein P6. Although these viral factories (VFs) were described half a century ago, with many observations that followed since, functional details of the condensation process and the properties and relevance of VFs have remained enigmatic. Here, we studied these issues in *Arabidopsis thaliana* and *Nicotiana benthamiana.* We observed a large dynamic mobility range of host proteins within VFs, while the viral matrix protein P6 is immobile, as it represents the central node of these condensates. We identified the stress granule (SG) nucleating factors G3BP7 and UBP1 family members as components of VFs. Similarly, as SG components localize to VFs during infection, ectopic P6 localizes to SGs and reduces their assembly after stress. Intriguingly, it appears that soluble rather than condensed P6 suppresses SG formation and mediates other essential P6 functions, suggesting that the increased condensation over the infection time-course may accompany a progressive shift in selected P6 functions. Together, this study highlights VFs as dynamic condensates and P6 as a complex modulator of SG responses.

**IN A NUTSHELL**

**Background:** Regulating RNA abundance and its availability for translation is one of the main struggles between virus and host during plant virus infections. The pararetrovirus *Cauliflower mosaic virus* (CaMV) shields its nucleic acids, proteins, and particles from its host antiviral pathways by establishing large, amorphous condensates, termed viral factories. These condensates consist mainly of 1 multifunctional viral protein, the P6 protein. We have previously found that host RNA decapping proteins, canonically associated with phase-separated RNA granules, localize within these viral factories, and aid in the translation of viral RNA. The interplay between virus replication and RNA granule biology is conserved among eukaryotes and likely represents an ancient mechanism.

**Question:** Our first aim was to further characterize the viral factory of CaMV and observe the behavior of host proteins within these condensates. Our second aim was to dissect the interaction of the viral P6 protein with RNA granules and RNA granule proteins, as well as its effect on translation in the context of condensation.

**Findings:** Unlike the rigid P6 matrix, RNA granule proteins can rapidly shuffle between viral factories and the surrounding cytoplasm. Several RNA granule proteins can bind viral RNA; however, their binding capacities seem to be specific for certain viral RNA species. Stress granule proteins preferentially bind to the noncoding highly abundant *8s* RNA, which may dampen stress granule responses in the plant. The P6 protein, in contrast, strongly localizes to stress granules and hinders their establishment due to its remarkable ability to enhance global translation levels. Importantly, the efficiency with which P6 exerts its functions is in part coupled to its condensation level, which likely represents a self-attenuation mechanism of CaMV during prolonged infections.

**Next steps:** We have merely scratched the surface of the viral factory-localized host proteome and RNAome. Are there common features of the plant proteins and RNAs that accumulate within the viral factories? Elucidating which host factors are co-opted by the virus will further our understanding of virus disease in plants.

## Introduction

The post-transcriptional fate of mRNA is controlled by an extensive network that mediates RNA processing, translation, and ultimately degradation (Chantarachot and Bailey-Serres 2018). An intriguing feature is the compartmentalization of many of these processes in membrane-less condensates termed RNA granules, including nucleoli, Cajal bodies, and paraspeckles in the nucleus and processing bodies (PBs) and stress granules (SGs) in the cytoplasm (Spector 2006). Studies on the physical properties of RNA granules suggest that they assemble through liquid–liquid phase separation (LLPS), a process influenced by RNA–RNA interactions, as well as RNA interactions with protein low-complexity or prion-like domains (Uversky 2017; Alberti et al. 2019; Youn et al. 2019; Boncella et al. 2020). RNA granules can assemble in a matter of minutes upon stress induction and disperse just as quickly when translationally favorable conditions return (Weber et al. 2008). This makes them a highly adaptable environment that offers possibilities for rapid and diverse molecular crowding and compartmentalization.

The most extensively studied cytoplasmic RNA granules in plants and other organisms are SGs and PBs, which are both implicated in the transient storage of nontranslating RNA (Kedersha and Anderson 2007; Decker and Parker 2012; Chantarachot and Bailey-Serres 2018; Guzikowski et al. 2019). An anticorrelation between ribosome- and SG-associated mRNAs was established in animals and plants (Sorenson and Bailey-Serres 2014; Khong et al. 2017). Despite enormous progress in the field, the functional importance of the actual assembly into microscopically visible RNA granules for mRNA regulation is still largely unclear (Guzikowski et al. 2019). Both SGs and PBs have the capacity to influence mRNA translation, storage, and decay, as well as protein signaling, and also serve as protective refuges during stress. Their formation is induced by RNA release from polysomes triggered by abiotic or biotic stress (Riggs et al. 2020). Despite representing distinct structures, they also share protein components and appear to even fully overlap under specific conditions (Buchan et al. 2013; Youn et al. 2018; Frydryskova et al. 2020), supporting the presence of a dynamic interface.

In the “mRNA cycle” model (Buchan and Parker 2009), mRNA movement between SGs, PBs, and ribosomes is considered to be both dynamic and bidirectional. Notably, the efficiency by which RNAs enter SGs is highly variable but is positively correlated with RNA length and poor translation (Khong et al. 2017). Viral RNAs can be long and polycistronic, making them a target of antiviral translational responses and SG regulation. Indeed, there are numerous examples of animal viruses being targeted by SGs (Poblete-Duran et al. 2016), and these RNA granules would appear to be an integral part of the host's antiviral defense, as well as a major target for manipulation by viral effectors (Lloyd 2016; Poblete-Duran et al. 2016; Miras et al. 2017; Pooggin and Ryabova 2018; Jaafar and Kieft 2019; Stern-Ginossar et al. 2019). The extent to which plant virus infections involve the SG pathway remains largely elusive. UBP1 (oligouridylate-binding protein 1), represented by the RBP45/RBP47/UBP1 family in Arabidopsis (*Arabidopsis thaliana*), is the closest homolog of the mammalian SG nucleation factor TIA-1 (T-cell intracellular antigen 1) (Lorkovic and Barta 2002; Weber et al. 2008; Gutierrez-Beltran et al. 2021), and was found to assemble in noncanonical RNA granules and to suppress the translation of Potato virus A RNA (Hafren et al. 2015). A second report suggested that 2 unrelated plant viral proteins can suppress SG formation through their interactions with G3BP (Ras-GAP SH3 domain-binding protein) (Krapp et al. 2017), another core SG nucleating factor (Abulfaraj et al. 2018; Yang et al. 2020). Assuming a similar codependence of plant viruses with SGs as observed for animal viruses, there is a great need to uncover the viral proteins that interact with SGs in plants.

*Cauliflower mosaic virus* (CaMV) is a double-stranded DNA pararetrovirus that replicates in large amorphous cytoplasmic inclusion bodies referred to as viral factories (VFs). These VFs have been described as nonmembranous electron-dense protein/RNA-rich structures like RNA granules. Even though VFs are considered to be functionally associated with viral genome replication, translation and particle assembly (Schoelz and Leisner 2017), our current understanding of these major structures and their dynamic functions is limited. The main component of VFs is the viral protein P6. P6 interacts with all other CaMV proteins (Himmelbach et al. 1996; Hapiak et al. 2008; Lutz et al. 2012), as well as a multitude of host proteins, to exert its many functions. One of the key functions of P6 is to regulate viral translation, a process involving physical interactions with ribosomal proteins, namely L13 (Bureau et al. 2004), L18 (Leh et al. 2000), and L24 (Park et al. 2001) and translational regulators eIF3g (eukaryotic translation initiation factor 3) (Park et al. 2001) and TOR (target of rapamycin) (Schepetilnikov et al. 2011). However, the spatial relationship between VFs and P6 in association with the translation machinery is still vague but could occur at the surfaces of VFs, as these structures were found to be decorated by ribosomes (Shepherd 1976).

We are interested in the dynamics and functions of CaMV VF-associated components and recently determined that the PB components DCP5 (decapping 5), LSM1a (like SM-protein and VCS (varicose) localize to VFs and support viral translation (Hoffmann et al. 2022). This raised the question of the possible analogy between RNA granules and VF matrixes. In the current study, we addressed this issue by studying plant responses to CaMV and identified and characterized SG components as factors in VFs.

## Results

### P6 forms immobile matrixes of VFs and self-condensates

**Figure 1.**
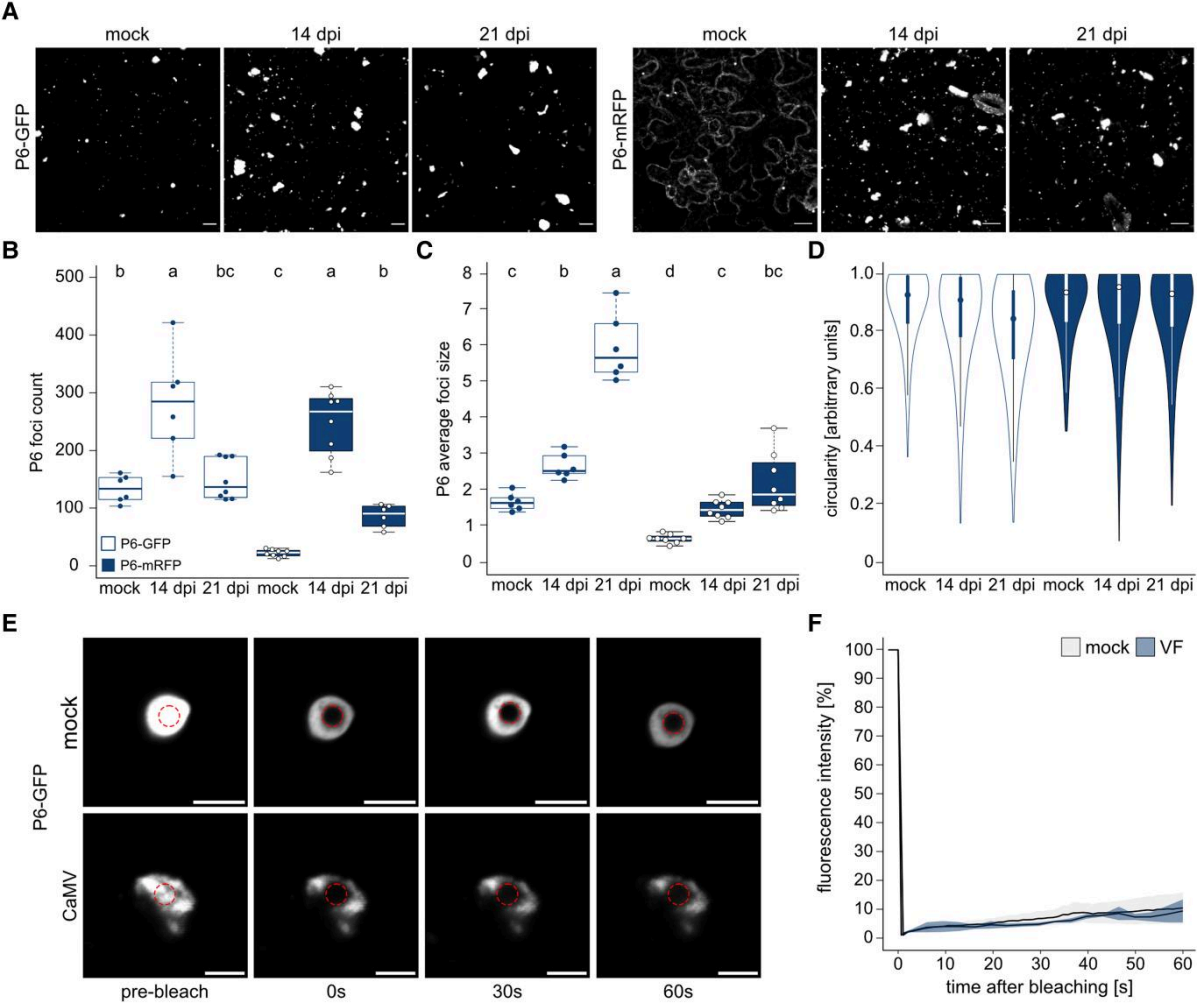
P6 establishes an immobile matrix in VFs. **A)** Maturation of P6 foci during an infection time-course (noninfected, 14 and 21 dpi) with CaMV was followed in Arabidopsis P6-GFP and P6-mRFP marker lines by microscopy. Representative images are confocal Z-stack projections (scale bars = 10 *µ*m). **B)** The number of P6-GFP and -mRFP foci in 100 × 100 *µ*m^2^ at timepoints corresponding to **A)**. Counts were obtained with a custom ImageJ pipeline using 6 to 8 replicate images. **C)** Average size of P6 foci in stacks corresponding to **B)**. Values were calculated from 6 to 8 replicates with a custom ImageJ pipeline. **D)** Circularity distribution of P6 foci at each time point, as determined by ImageJ circularity masking. **E)** FRAP analysis of P6-GFP in mock conditions (*n* = 7) and in VFs at 21 dpi (*n* = 8). The photobleached region is indicated by a red circle. Scale bars = 5 *µ*m. **F)** Normalized fluorescence intensities in FRAP analysis corresponding to **(E)** are plotted against time after bleaching. Solid lines represent mean, shades denote ±Sd. Letters in **(B)** to **(C)** indicate statistical groups determined by 1-way ANOVA followed by Tukey's HSD test (α = 0.05). For boxplots: the box represents the IQR, the solid lines represent the median. Whiskers extend to a maximum of 1.5× IQR beyond the box. For Violinplots: the box represents the IQR, the solid lines represent the median. Whiskers extend to a maximum of 1.5× IQR beyond the box. Violin shows the kernel probability density of the data.

VFs are essential structures in CaMV infection and are largely built by P6, but the aggregation dynamics and mobility of P6 within VFs are unclear. We monitored VF size and changes in shape along the course of infection using the transgenic Arabidopsis marker lines 35S:P6-GFP and 35S:P6-mRFP. In the absence of infection, P6-GFP formed numerous foci spanning a broad size range similar to that observed previously (Harries et al. 2009), while P6-mRFP was mainly soluble and formed much fewer, smaller, and more uniformly sized foci (Fig. 1, A to C). When P6 marker lines were infected with CaMV, both P6-GFP and P6-mRFP relocalized to mark the characteristic large amorphous VFs that formed during infection (Fig. 1A). Quantification showed that the number of foci decreased while the size and irregularity increased between 14- and 21-days postinfection (dpi) (Fig. 1, B to D). This can be explained by the fusion of smaller P6 foci to eventually mature into few but large VFs per cell, in accordance with previous interpretations (Shepherd 1976).

While having a markedly different degree of condensation under uninfected conditions, P6-GFP and P6-mRFP behaved similarly, as they abundantly accumulated in VFs during infection. To estimate the mobility of P6 in these structures, we performed fluorescence recovery after photobleaching (FRAP) analysis of P6-GFP- and P6-mRFP-tagged VFs following CaMV infection as well as P6-GFP condensates in the absence of infection (Fig. 1, E and F; Supplemental Fig. S1). P6 was nearly static, with very little recovery observed under all conditions, supporting the notion that P6 is largely immobile in VFs and forms a robust VF matrix.

### Core SG components localize to VFs

**Figure 2.**
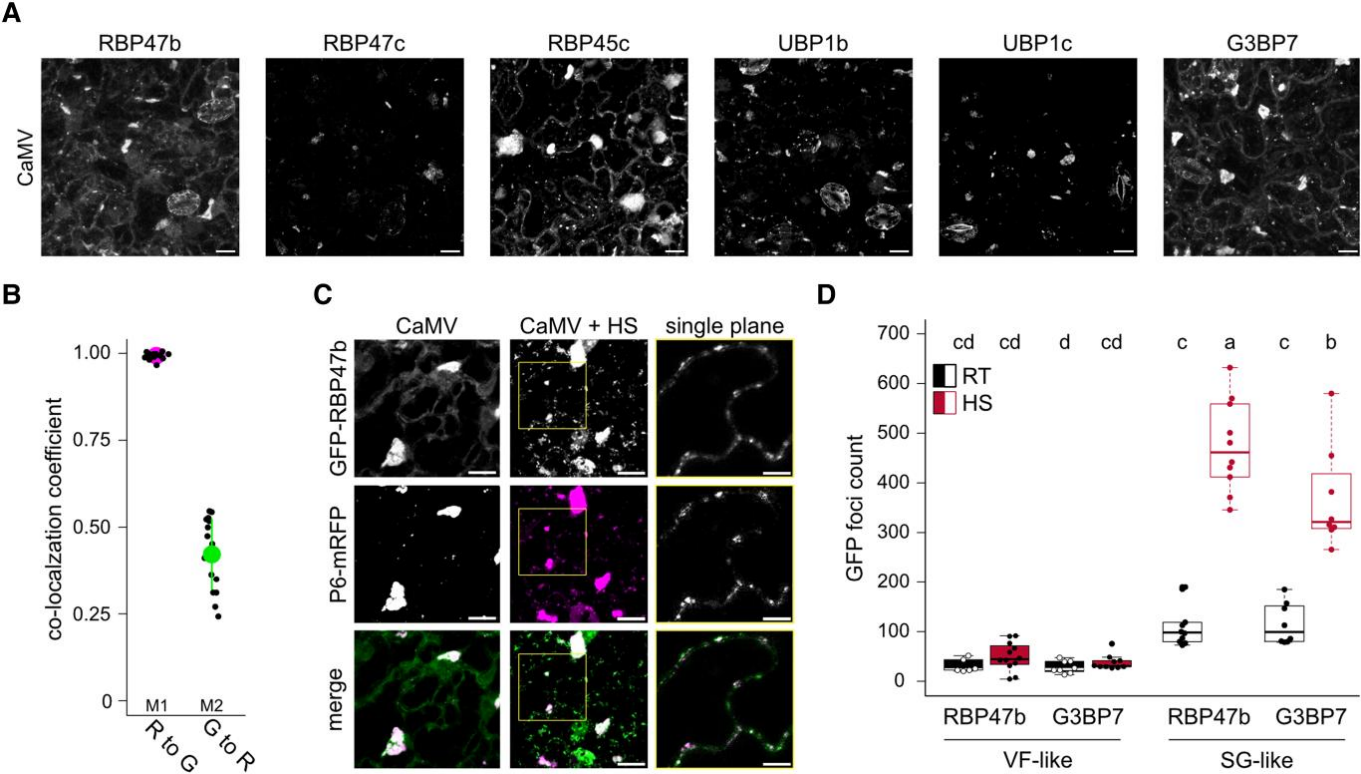
SG proteins localize to VFs. **A)** Localization of canonical SG markers in Arabidopsis at 21 dpi with CaMV. Representative images are confocal Z-stack projections (Scale bars = 10 *µ*m). **B)** Mander's colocalization coefficients of GFP-RBP47b (G) and P6-mRFP (R) at 21 dpi with CaMV. Values were calculated from 16 Z-stacks using the ImageJ plugin JACoP. **C)** Colocalization of GFP-RBP47b and P6-mRFP 21 dpi with CaMV after 30 min of HS. Representative images are confocal Z-stack projections (scale bars = 10 *µ*m). The insets from CaMV + HS (yellow squares) are shown in the right column and represent single plain images (scale bars = 5 *µ*m). **D)** Foci counts in 100 × 100 *µ*m^2^ in infected tissues for GFP-RBP47b and GFP-G3BP7. Foci were separated into SG-like (<2 *µ*m^2^) or VF-like (>2 *µ*m^2^). HS, heat shock; RT, room temperature. Counts were averaged from 10 replicate images with a custom ImageJ pipeline. Letters indicate statistical groups determined by 1-way ANOVA followed by Tukey's HSD test (α = 0.05).

We recently showed that PB components localize to VFs during CaMV infection (Hoffmann et al. 2022). Here, we further explored the nature of these condensates, particularly to what extent they resemble cytoplasmic RNA granules. To evaluate SG components in VFs, we established marker lines of canonical SG proteins, namely GFP-RBP45c, GFP-RBP47b, GFP-RBP47c, GFP-UBP1b, GFP-UBP1c, and GFP-G3BP7 in the Arabidopsis Col-0 background. The 6 markers localized in a diffuse nucleocytoplasmic pattern and assembled into microscopical foci upon heat stress (Supplemental Fig. S2A). In CaMV-infected tissue, however, all markers localized to the large amorphous foci characteristic of VFs in addition to similar sized SG-like foci observed in response to heat stress (Fig. 2A). By performing colocalization analysis using a double marker line expressing GFP-RBP47b and P6-mRFP, we further found that GFP-RBP47b indeed localizes to VFs, with almost all P6 signal comarked by GFP-RBP47b (Fig. 2, B and C, “M1”) and ∼40% of the total GFP-RBP47b signal present in P6 foci (Fig. 2, B and C, “M2”).

We also analyzed the previously described Arabidopsis eIF4A-GFP line, which is reported to assemble SGs in response to heat stress (Hamada et al. 2018). We confirmed that eIF4A-GFP formed numerous foci in the cytoplasm upon heat shock (HS); however, it did not localize to VFs upon CaMV infection (Supplemental Fig. S2B). Intriguingly, when CaMV-infected eIF4A-GFP plants were subjected to heat stress, this marker did indeed enter VFs, which were morphologically different from the HS-induced foci observed in noninfected plants (Supplemental Fig. S2B). These results expand the repertoire of VF localized RNA granule proteins. However, the specific absence of eIF4A from VFs points to possible selectivity of SG components by VFs, as we observed the specific absence of the PB component DCP1 (decapping 1) in these structures (Hoffmann et al. 2022). Alternatively, eIF4A could be a conditional SG component.

To evaluate this issue, we chose arsenite treatment as another commonly used stressor leading to stalled translation and SG assembly (Bernstam and Nriagu 2000; Sorenson and Bailey-Serres 2014). We detected polysome disassembly in response to both heat stress and arsenite, although this response was milder for arsenite (Supplemental Fig. S2C). The quantitative difference on polysomes was also reflected by the formation of more numerous SGs in response to heat stress vs. arsenite in both the G3BP7 and RBP47b marker lines (Supplemental Fig. S2, D and E). Notably, eIF4A did not form SG foci upon arsenite stress (Supplemental Fig. S2, D and E), suggesting that eIF4A is a conditional SG component and that SG component composition within VFs in ambient temperatures is closer to that induced by arsenite vs. heat stress.

It is interesting that CaMV induces the formation of SG-like foci in addition to VFs, despite the enhanced translation levels in infected tissues (Fig. 2, A and D) (Hoffmann et al. 2022), which is opposite to the canonical anticorrelation between these 2 processes. Importantly, the number of SG-like foci increased substantially in infected tissues after HS, while the number of VF-like foci remained constant (Fig. 2D), suggesting that infected tissue is competent for de novo SG assembly. However, despite the resemblance of these newly assembled heat-induced SGs to those observed in noninfected tissues, a closer inspection showed that they frequently also contained P6 (Fig. 2C) and may therefore be functionally diverted and controlled by CaMV. Taken together, based on their recruitment of several SG proteins, we conclude that VFs act as sponges for RNA granule proteins during CaMV infection, with some selectivity and environmental dependence, as judged from eIF4A.

### VFs, unlike PBs and SGs, do not depend on polysomal mRNA supply

**Figure 3.**
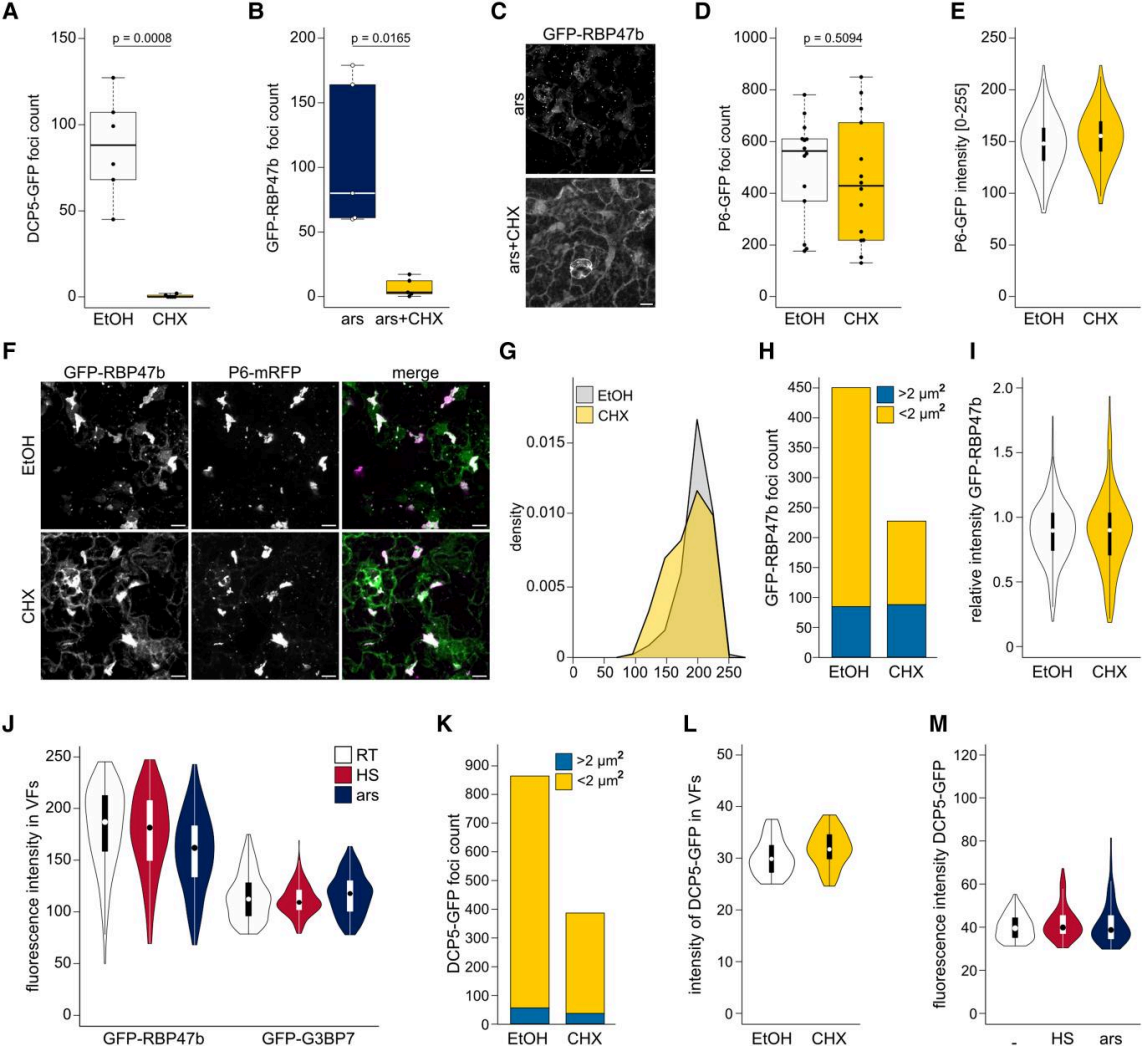
RNA granule components in VFs are unresponsive to SG/PB inhibition or induction. **A)** DCP5-GFP foci counts in 100 × 100 *µ*m^2^ after EtOH (control) or 200 *µ*M CHX treatment for 2 h. Counts were averaged from 6 replicates. **B)** GFP-RBP47b foci counts in 100 × 100 *µ*m after 1 mM arsenite + EtOH and 1 mM arsenite +200 *µ*M CHX treatment for 2 h. Counts were averaged from 6 replicates. **C)** Representative image of the GFP-RBP47b marker line after induction by arsenite (ars; upper panel) and additional treatment with CHX (lower panel) corresponding to **(B)** (scale bars = 10 *µ*m). **D)** P6-GFP foci counts in 100 × 100 *µ*m^2^ after EtOH or 200 *µ*M CHX treatment for 2 h. Counts were averaged from 14 replicates. **E)** Fluorescent intensity of P6-GFP foci after EtOH and CHX treatment in **(D)**. Violin plots represent counts of 7,513 (EtOH) and 5,839 (CHX) foci. **F**) Representative image of GFP-RBP47b and P6-mRFP double marker line 21 dpi with CaMV after treatment with either EtOH (upper panel) or 200 *µ*M CHX (lower panel) for 2 h (Scale bars = 10 *µ*m). **G)** Frequency diagram of P6-mRFP signal intensity in VFs after EtOH or CHX treatment. The *x* axis denotes the fluorescence intensity, the *y* axis denotes the counts in each bin (bin width = 25). The same imaging set up was used as in **(F)** to **(I)**. **H)** GFP-RBP47b total foci count split between SG-like foci (<2 *µ*m^2^) and VF-like foci (>2 *µ*m^2^) after EtOH or CHX treatment. The same imaging set up was used as in **(F)**, **(G)**, and **(I)**. **I)** Relative intensity of GFP-RBP47b compared to P6-mRFP within VFs after EtOH or CHX treatment. The same imaging set up was used as in **(F)** to **(H)**. **J)** Fluorescent intensity of GFP-RBP47b and GFP-G3BP7 foci in RT, after 30 min HS at 38 °C, or after 1 mM arsenite treatment for 2 h (ars); *n* = 135 to 153 VFs in each condition. **K)** DCP5-GFP total foci count split between SG-like foci (<2 *µ*m^2^) and VF-like foci (>2 *µ*m^2^) after EtOH or 200 *µ*M CHX treatment for 2 h. **L)** Fluorescent intensity of DCP5-GFP in VFs after EtOH (*n* = 57) or CHX treatment (*n* = 38) as in **(K)**. **M)** Fluorescent intensity of DCP5-GFP in VFs in ambient temperatures, after 30 min HS at 38 °C or 1 mM arsenite treatment for 2 h; *n* = 82 to 95 VFs in each condition. Statistical significance for **(A)**, **(B)**, and **(D)** was calculated by Welch Two Sample *t*-test. For boxplots, the box represents the IQR, the solid lines represent the median. Whiskers extend to a maximum of 1.5× IQR beyond the box. For violinplots, the box represents the IQR, the solid lines represent the median. Whiskers extend to a maximum of 1.5× IQR beyond the box. Violin shows the kernel probability density of the data.

A hallmark characteristic of canonical PBs and SGs is their sensitivity to cycloheximide (CHX), a drug that inhibits translational elongation and locks ribosomes on RNA; this inhibits ribosome runoff, thereby reducing the availability of mRNA for granulation. Accordingly, we observed both the disassembly of DCP5-GFP-positive PBs and the prevention of arsenite-induced GFP-RBP47b SG formation in the presence of CHX (Fig. 3, A to C). In contrast, this treatment had no evident effect on the number or signal intensity of P6-GFP foci (Fig. 3, D and E). This suggests that P6 condensates that form in the absence of infection is not as dependent on mRNA supply from ribosomes as canonical mRNA granules. We used the double marker line GFP-RBP47b and P6-mRFP to test the susceptibility of SG proteins within VFs to CHX. Like P6 condensates, VFs appeared to be unaffected by CHX treatment (Fig. 3, F and G). While small cytoplasmic foci readily disappeared in infected tissue after CHX treatment, the large VFs persisted (Fig. 3H) and were still marked by GFP-RBP47b with the same signal intensity (normalized to P6) as the EtOH control (Fig. 3I). We then used the SG-inducing conditions heat and arsenite in conjunction with fluorescence intensity monitoring of GFP-tagged RBP47b or G3BP7 in VFs but did not detect any differences between the treatments (Fig. 3J). We observed the same behavior with the PB marker DCP5, where CHX treatment diminished canonical PB but not VF formation (Fig. 3K). Likewise, neither CHX, arsenite, nor heat affected the amount of DCP5 fluorescence in VFs (Fig. 3, L and M). Together, these findings suggest that VFs, along with SG and PB components, do not exhibit a similar interdependence on mRNA channeling from the translation machinery as canonical SGs or PBs.

### RNA granule proteins remain highly mobile in VFs

**Figure 4.**
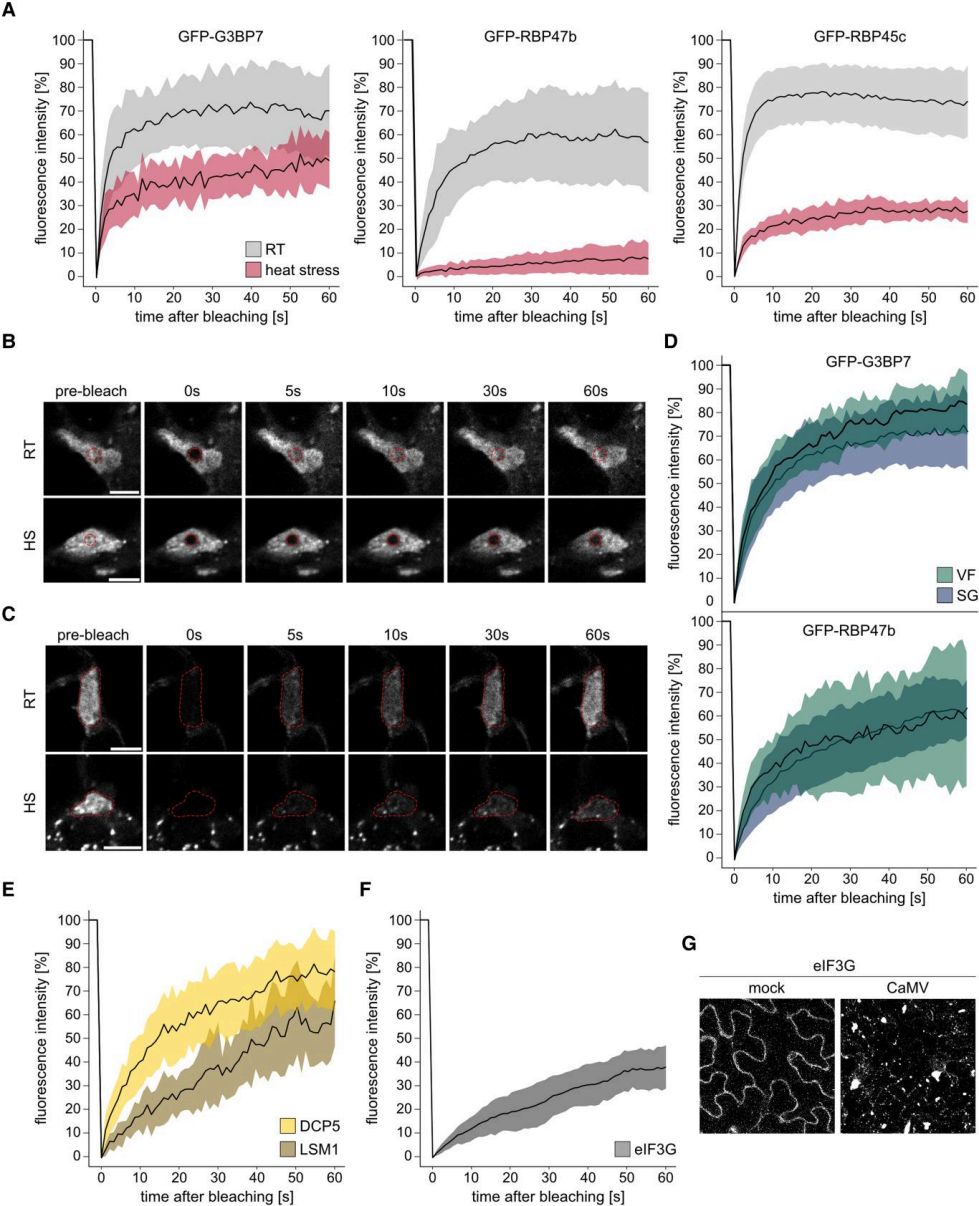
RNA granule proteins shuttle rapidly within VFs and their surroundings. **A)** FRAP analysis of the indicated proteins in VFs at 21 dpi at ambient temperatures (RT) or after 30 min of 38 °C HS. Normalized fluorescence intensities are plotted against time after photobleaching; *n* = 5 to 13. **B)** Representative image series from FRAP analysis of GFP-RBP47b after photobleaching corresponding to RT and HS in **(A)**. Photobleached region is indicated by a circle. Scale bars = 5 *µ*m. **C)** Representative image series from FRAP analysis of GFP-G3BP7 after photobleaching the whole VF at RT or after 30 min of 38 °C HS. Photobleached region is indicated by an outline. Scale bars = 5 *µ*m. **D)** FRAP analysis of the indicated proteins in VFs and SGs at 21 dpi after 2 h of 1 mM arsenite treatment. Normalized fluorescence intensities are plotted against time after bleaching; *n* = 11/13 for VFs and 17/31 for SGs. **E)** FRAP analysis of DCP5 (*n* = 35) and LSM1a (*n* = 16) proteins in VFs at 21 dpi at ambient temperatures. Normalized fluorescence intensities are plotted against time after bleaching. **F)** FRAP analysis of eIF3g in VFs at 21 dpi at ambient temperatures. Normalized fluorescence intensities are plotted against time after bleaching; *n* = 7. **G)** Localization of eIF3g-GFP under uninfected mock conditions and 21 dpi with CaMV. Representative images are composed of confocal Z-stacks (scale bars = 10 *µ*m). **A, D to F)** Solid lines represent mean, shades denote ±Sd.

The insensitivity of SG proteins to CHX regarding their association with VFs suggests they might play a role other than the translational repression of transcripts. Furthermore, the unconventional overlap of PB and SG components points to an aberrant RNA granule character that is usually associated with the relative immobility of RNA granule proteins compared to SGs and PBs in liquid phase (Frydryskova et al. 2020). However, FRAP analysis showed that G3BP7, RBP47b, and RBP45c were all highly mobile within VFs and recovered within 5 to 10 s after bleaching (Fig. 4, A and B). Furthermore, these proteins were also constantly exchanged with the surrounding cytoplasm, as indicated by abundant fluorescence recovery after bleaching the whole VF (Fig. 4C). Thus, SG proteins are highly dynamic within VFs and show comparable FRAP recovery rates to those observed in mammalian SGs for G3BP7 and RBP47b homologs G3BP1 and TIA-1, respectively (Kedersha et al. 2000, 2005).

To further explore the analogy between VFs and canonical SGs, we compared their component dynamics during heat and arsenite stress. Upon heat treatment, G3BP7 still recovered quickly in both VFs and SGs, although a fraction of the protein became immobile (Fig. 4A; Supplemental Fig. S3A). In contrast, RBP47b, RBP45c, and eIF4A were largely immobile within VFs and SGs in response to heat stress (Fig. 4, A and B; Supplemental Fig. S3A). We conclude that SG component dynamics are different in VFs under ambient conditions compared to heat-induced SGs, but VFs undergo an intriguing transformation in this direction when subjected to heat stress, as already supported by the conditional eIF4A targeting (Supplemental Fig. S2B).

Considering that eIF4A foci assemble only upon heat but not arsenite stress, in contrast to G3BP7 and RBP47b, and that SGs induced by heat and arsenite can differ in many aspects (Frydryskova et al. 2020), we also performed FRAP analysis of these components after arsenite treatment. Notably, both proteins remained mobile and recovered quickly within both VFs and SGs, in contrast to their behavior after heat stress (Fig. 4D). The 2 canonical PB proteins LSM1a and DCP5 were also highly mobile within VFs, although LSM1a recovery was slower, and the protein had a larger immobile phase than DCP5 (Fig. 4E). The rapid shuttling of the tested RNA granule proteins within the VFs and between VFs and the cytoplasm suggests that large fractions of these proteins do not bind strongly to the immobile P6 matrix. We therefore used eIF3g, which is known to directly interact with P6 (Park et al. 2001), to evaluate its presence in VFs and, more importantly, to determine whether a direct interaction leads to a similar level of immobility to that observed for P6. eIF3g-GFP primarily localized to VFs and indeed displayed slower recovery than SG proteins but was still clearly mobile compared to P6 (Fig. 4, F and G). Altogether, we conclude that (i) RNA granule proteins are not rigidly bound to or aggregated within the immobile P6 phase, but shuttle between the VFs and their surroundings; (ii) under ambient conditions VFs resemble arsenite- but not heat-treated SGs in terms of composition and mobility; and (iii) VFs adopt the characteristic dynamics of heat-stressed SGs upon exposure to this stress.

### 35S genomic viral RNA binds to PB components while avoiding SG components in planta

**Figure 5.**
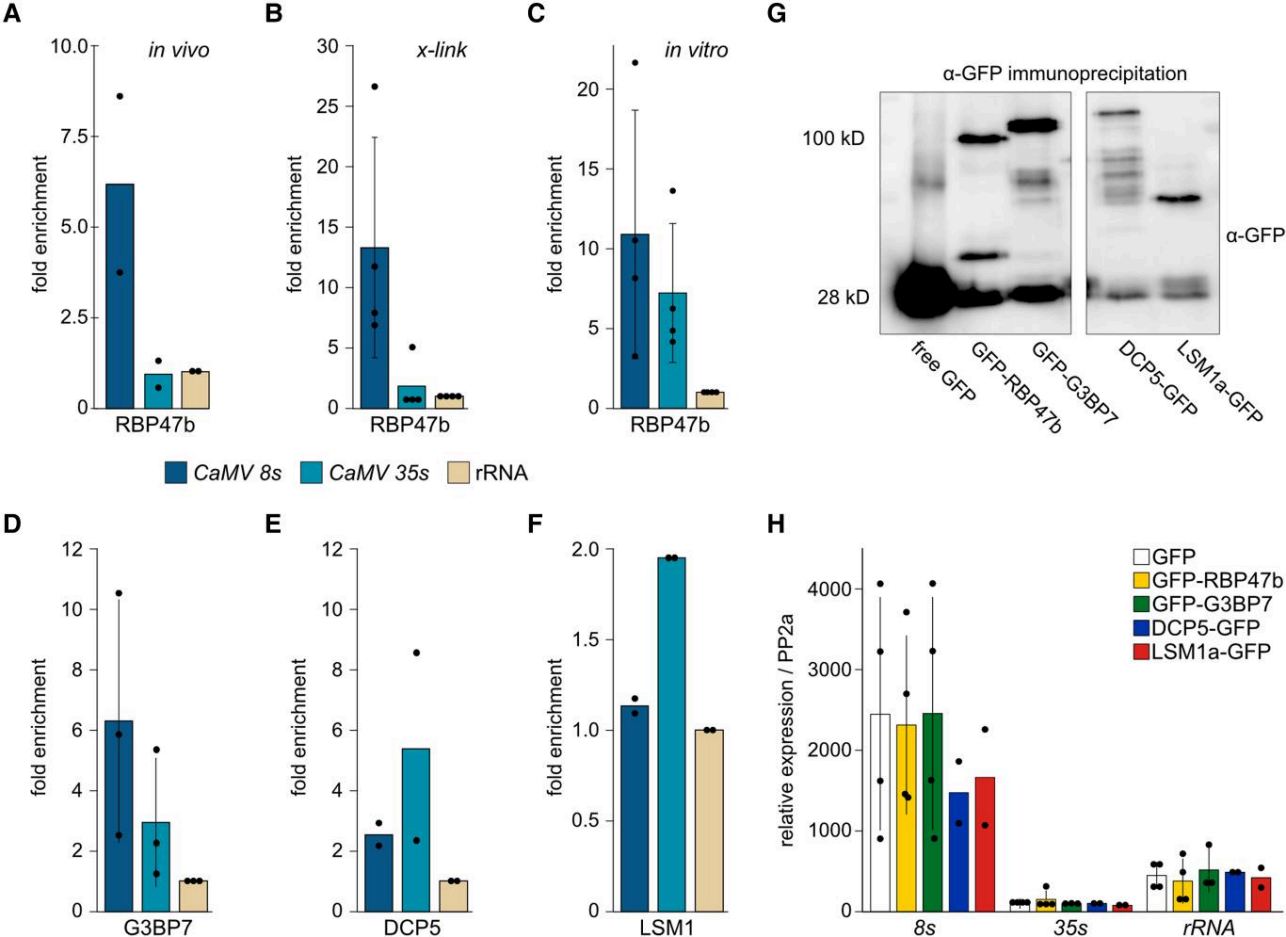
SG and PB proteins strongly associate with different viral RNAs. **A)** Fold enrichment of viral RNAs in native RIPA from GFP-RBP47b over free GFP-expressing plants using *ribosomal (r)RNA* for calibration. Data points represent independent experiments; *n* = 2 independent experiments. **B)** As in **(A)** but including an in planta FA cross-linking step prior to RIPA; *n* = 4 independent experiments. **C)** Fold enrichment of viral RNAs in an in vitro RIPA with GST-RBP47b over the GST control using *rRNA* for calibration. Data points represent independent experiments; *n* = 4 independent experiments. **D, E, and F)** Fold of enrichment of viral RNAs in FA cross-linked RIPAs from GFP-G3BP7 **(G)**, DCP5-GFP **(H)**, and LSM1a-GFP **(I)** over free GFP expressing plants using *rRNA* for calibration. Data points represent independent experiments; *n* = 2 to 3 independent experiments. **G)** Immunoblot analysis using anti-GFP to verify capture of baits in the RIPAs of GFP, RBP47b, G3BP7, DCP5, and LSM1a; *n* = 2 to 3 independent experiments. Ponceau S (PS) staining served as loading control. **H)** Relative expression of viral RNAs and *rRNA* in input fractions of RIPA samples normalized to housekeeping gene *PP2a*; *n* = 4 independent experiments. Bars indicate mean of independent experiments, error bars denote ±Sd. Dots indicate single experiments.

A primary function of SG and PB components is RNA regulation through RNA binding, prompting us to address if any of these components could be detected in association with viral RNA during infection. First, we infected GFP-RBP47b and free GFP control Arabidopsis plants and harvested symptomatic tissue at 21 dpi for GFP-based coprecipitation. We detected copurification of the viral protein P6 but not P4 using immunoblot analysis (Supplemental Fig. S4), and interestingly also the viral *8S* leader RNA but not the protein-coding full viral *35S* RNA or control *rRNA* (Fig. 5A). To reduce the risk of disassociation during isolation, we included an in planta formaldehyde (FA) cross-linking step, which led to slightly better capture of *8S* but still no detectable enrichment of *35S* (Fig. 5B). However, an in vitro association assay performed largely according to Dember et al. (1996) showed that GST-RBP47b could bind to *35S* RNA with comparable efficiency to *8S*, as both were highly enriched over the GST control and the background *rRNA* control (Fig. 5C). Next, we extended the in planta RNA-immunoprecipitation assay (RIPA) to also include G3BP7, DCP5, and LSM1a. This revealed that G3BP7 (like RBP47b) also associated more strongly with *8S* than with *35S* RNA, whereas both PB components associated more strongly with *35S* than with *8S* (Fig. 5, D to F). Immunoblot analysis verified the capture of the baits in RIPA (Fig. 5G). Moreover, the total transcript levels in the assay (Fig. 5H) suggested that while SG components may associate with *8S*/*35S* in a somewhat total quantity-dependent manner, PB components DCP5 and LSM1a select for (and thus appear to be more specific regulators of) *35S* RNA.

### Overexpression of UBP1 family members reduces CaMV infectivity

**Figure 6.**
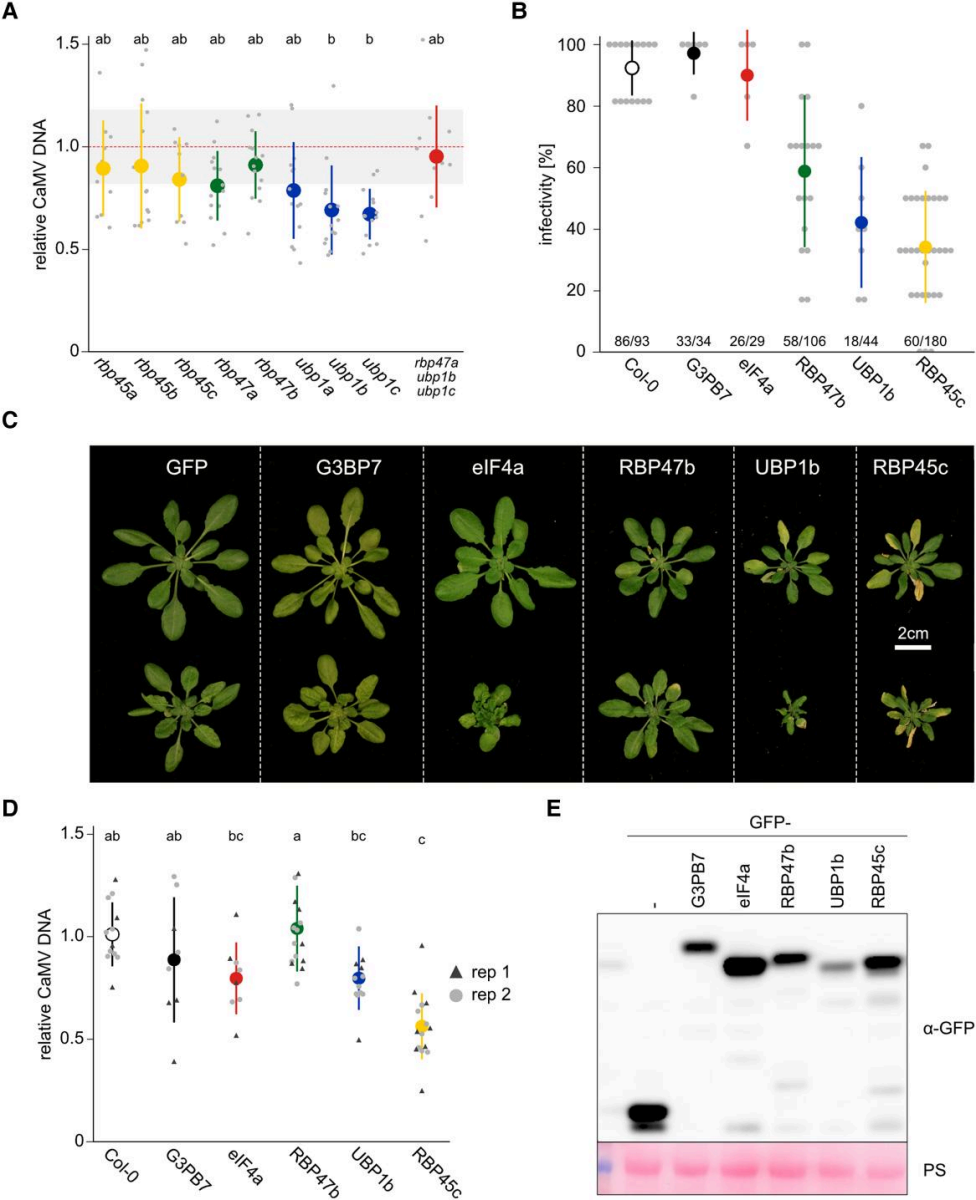
Overexpression (OEX) of UBP1 family members reduces CaMV infectivity. **A)** Viral DNA accumulation in systemic leaves of the indicated genotypes at 21 dpi, as determined by RT-qPCR. Average is depicted by large dots, and replicates by small dots (*n* = 12), error bars denote ±Sd. Values are relative to Col-0 plants and normalized to *18S* ribosomal DNA as the internal reference. Letters indicate statistical groups determined by 1-way ANOVA followed by Tukey's HSD test (α = 0.05). **B)** Infection success of CaMV scored at 21 dpi. Six plants were infected per pot and infectivity calculated as the fraction of systemically infected plants/total number of plants per pot. The total number of plants screened for each line is indicated below the graph. Average is depicted by large dots, and replicates by small dots, error bars denote ±Sd. **C)** Representative images of OEX lines of SG components not infected (upper panel) and 21 dpi with CaMV (lower panel). Scale bar = 2 cm. **D)** Viral DNA accumulation in systemic leaves of the indicated OEX lines at 21 dpi, as determined by RT-qPCR. Average is depicted by 1 large dot, and replicates by small symbols (*n* = 8 to 16), error bars denote ±Sd. Values are relative to Col-0 plants and normalized to *18S* ribosomal DNA as the internal reference. Letters indicate statistical groups determined by 1-way ANOVA followed by Tukey's HSD test (α = 0.05). **E)** Immunoblot analysis using anti-GFP to visualize GFP-fusion protein accumulation in OEX-lines. All tested lines expressed GFP-fusion proteins (GFP-). Free GFP (GFP-) was used as control line. PS staining served as loading control.

An outstanding question was whether SG components participate in CaMV infection, as previously established for PB components DCP5 and LSM1a (Hoffmann et al. 2022). The 9 members of the RBP47b gene family are largely uncharacterized (for a phylogenetic tree, see Sorenson and Bailey-Serres 2014), with knock-out phenotypes identified individually for *UBP1b* and *UBP1c* suggesting some nonredundant functions (Sorenson and Bailey-Serres 2014; McCue et al. 2012). We established a collection of Arabidopsis T-DNA insertion mutants in members of this gene family as well as a triple mutant (*rbp47a ubp1b ubp1c*) to address their importance for CaMV accumulation. However, these mutations had no evident effect, as even the slight reduction in CaMV accumulation initially observed in *ubp1b* and *ubp1c* was absent in the combinatorial triple mutant (Fig. 6A). Perhaps there is a high degree of redundancy within this gene family and their functions in CaMV infection, as several of them localize to VFs (Fig. 2A), or perhaps CaMV evades the antiviral properties of these proteins during infection.

Thus, we reasoned that overexpressing SG components might interfere with CaMV infection. To that end, we tested Arabidopsis overexpressor (OEX) lines of 5 SG marker proteins for CaMV infectivity. CaMV infection success was high in Col-0, G3BP7, and eIF4A but dropped substantially in RBP47b, UBP1b, and RBP45c (Fig. 6B). Interestingly, the latter 3 lines exhibited developmental phenotypes, including reduced growth and early senescence (Fig. 6C), which was previously observed in UBP1c OEX-lines (Sorenson and Bailey-Serres 2014). CaMV DNA accumulation in systemically infected OEX plants showed a general trend of reduction, but this was statistically significant only in RBP45c (Fig. 6D). Immunoblot analysis confirmed the accumulation levels of all tested proteins within the generated lines (Fig. 6E), which interestingly did not align quantitatively with the observed phenotypic severities. Taken together, while we currently lack a straight-forward system to analyze the general importance of the SG machinery in plant infection with CaMV, our results with OEX-lines suggest that SG components act as negative regulators of this process, in contrast to PB components, which aid infection (Hoffmann et al. 2022).

### P6 stabilizes polysomes and suppresses SG formation via eIF3g

**Figure 7.**
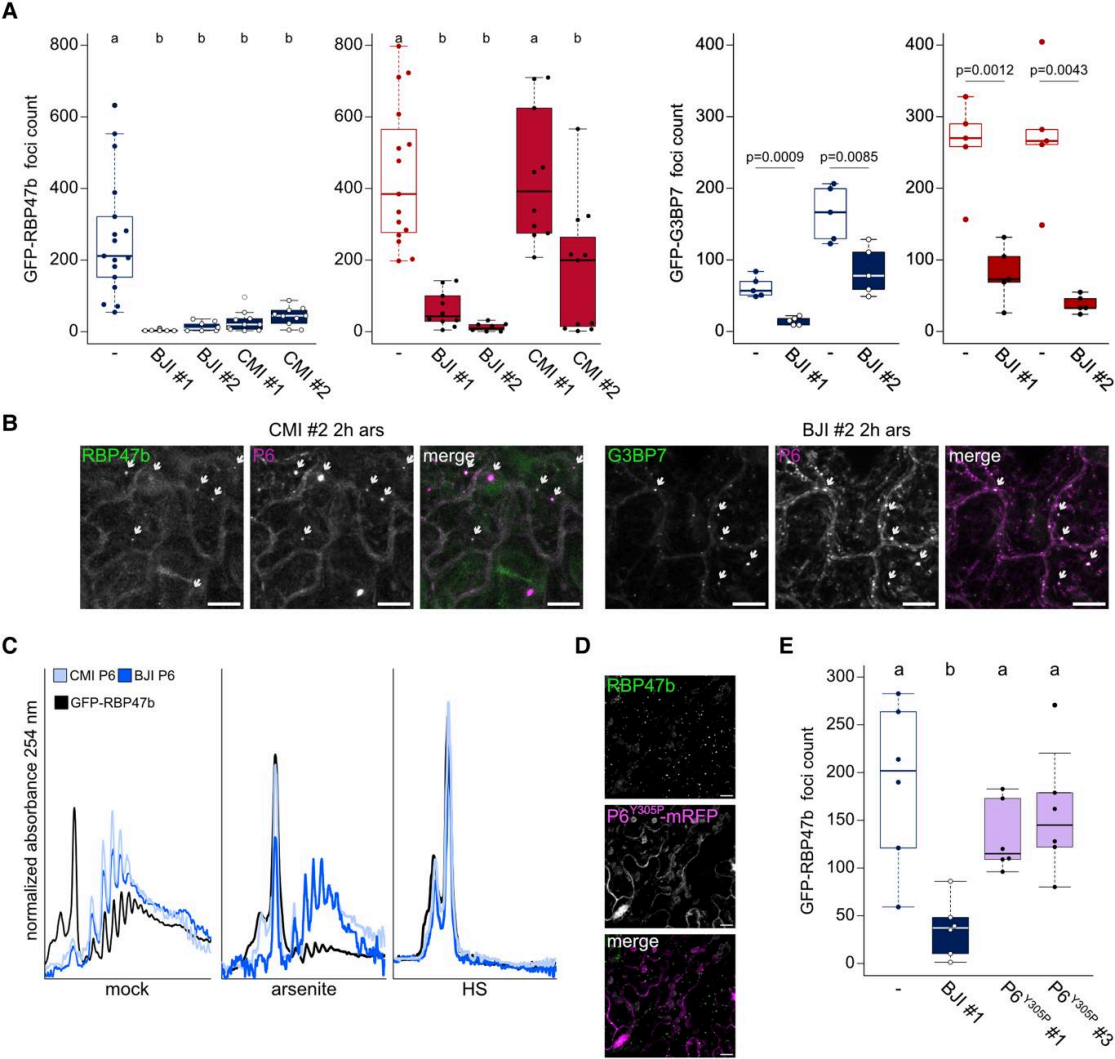
P6 inhibits SG formation. **A)** GFP-RBP47b and GFP-G3BP7 foci counts in double marker lines with P6-mRFP in 100 × 100 *µ*m^2^ regions. Counts were averaged from 10 to 15 replicates after 1 mM arsenite (ars) treatment for 2 h (left panel) or 30 min of 38 °C HS treatment (right panel). # denotes independent transgenic lines. The G3BP7 data for lines nos 1 and 2 were obtained separately and thus have individual controls (parental lines, marked with “-”) to their left; statistically significance differences for these groups were determined by Welch Two Sample *t*-test. **B)** Representative images of GFP-RBP47b and GFP-G3BP7 corresponding to **(A)** and **(B)**. Examples of condensates containing both the SG marker and P6 are marked by arrows (scale bars = 10 *µ*m). **C)** Polysome profiles of GFP-RBP47b with and without expression of P6s in untreated plants and after arsenite or heat treatment as in **(A)**. **D)** Representative images of GFP-RBP47b and P6Y305P-mRFP double-marker line (scale bars = 10 *µ*m). Note, the signal intensity of this mutant P6 was clearly lower, causing a partial bleed-through signal from chloroplasts in the P6 channel. **E)** GFP-RBP47b foci counts in double marker lines with P6Y305P-mRFP in 100 × 100 *µ*m^2^ regions. Counts were averaged from 6 replicates after arsenite treatment. Two independent lines were used (nos 1 and 2), “-” marks parental control line. **A, E)** Letters indicate statistical groups determined by 1-way ANOVA followed by Tukey's HSD test (α = 0.05). For boxplots: the box represents the IQR, the solid lines represent the median. Whiskers extend to a maximum of 1.5× IQR beyond the box.

As observed in Fig. 2C, P6 localized to potentially newly formed SGs upon heat stress in infected tissue, but whether these were truly de novo assemblies are unclear, and if this also occurs in the absence of infection is unknown. We challenged Arabidopsis marker-lines of GFP-RBP47b and GFP-G3BP7 expressing P6-mRFP with arsenite or heat stress, followed by quantification of SG-like foci assembly. Intriguingly, the P6-mRFP lines generally showed strongly reduced amounts of detectable Rbp47b and G3BP7 foci compared to their parental lines (Fig. 7A), except for CMI line no. 1 during heat stress. Overall, the GFP-RBP47b signal appeared weak in the P6 lines, but immunoblot analysis did not detect any clear differences in GFP-RBP47b or GFP-G3BP7 levels compared to the control (Supplemental Fig. S5). Notably, the residual SG foci that still formed were frequently colabeled by P6 (Fig. 7B), supporting the notion that P6 can also localize to SGs in the absence of infection.

CaMV P6 is a master regulator of *35S* RNA translation (Pooggin and Ryabova 2018), including mechanisms of translation reinitiation together with eIF3g (Park et al. 2001). Excitingly, we found that the polysome-to-monosome ratios were clearly higher in P6-mRFP lines compared to the parental control (Fig. 7C), suggesting that P6 might have a global impact on polysomes and could contribute to the observed increase in polysomes during CaMV infection (Park et al. 2001; Hoffmann et al. 2022). Moreover, arsenite treatment strongly reduced polysome levels in the parental control, whereas the P6-mRFP lines largely resisted this treatment, despite an evident increase in monosome levels. Moreover, heat stress was sufficient to fully disassemble the polysomes in all lines (Fig. 7C). While it is conceivable that polysome stabilization by P6 contributes to the reduced SG formation upon arsenite treatment, the strong inhibition of SG formation despite full polysome disassembly during heat stress points toward an additional uncoupled mechanism. We pursued the importance of P6 translation reinitiation mechanisms in inhibiting SG formation by establishing Arabidopsis GFP-RBP47b lines expressing P6-mRFP with tyrosine 305 (P6Y305P) swapped to proline, a mutation that disrupts the essential eIF3g interaction and translation transactivation in vitro (Park et al. 2001) but retains, e.g. the suppression of salicylic acid responses (Love et al. 2012). The Y305P mutation compromised P6-induced suppression of SG formation in response to arsenite, abolished colocalization, and failed to show any self-condensates (Fig. 7, D and E). Together, these results establish the capacity of P6 to counteract SG formation during stress, its prominent localization to SGs, and the potential importance of the eIF3g interaction in these processes.

### SG inhibition and trans-activation can be uncoupled and are reduced by P6 condensation

**Figure 8.**
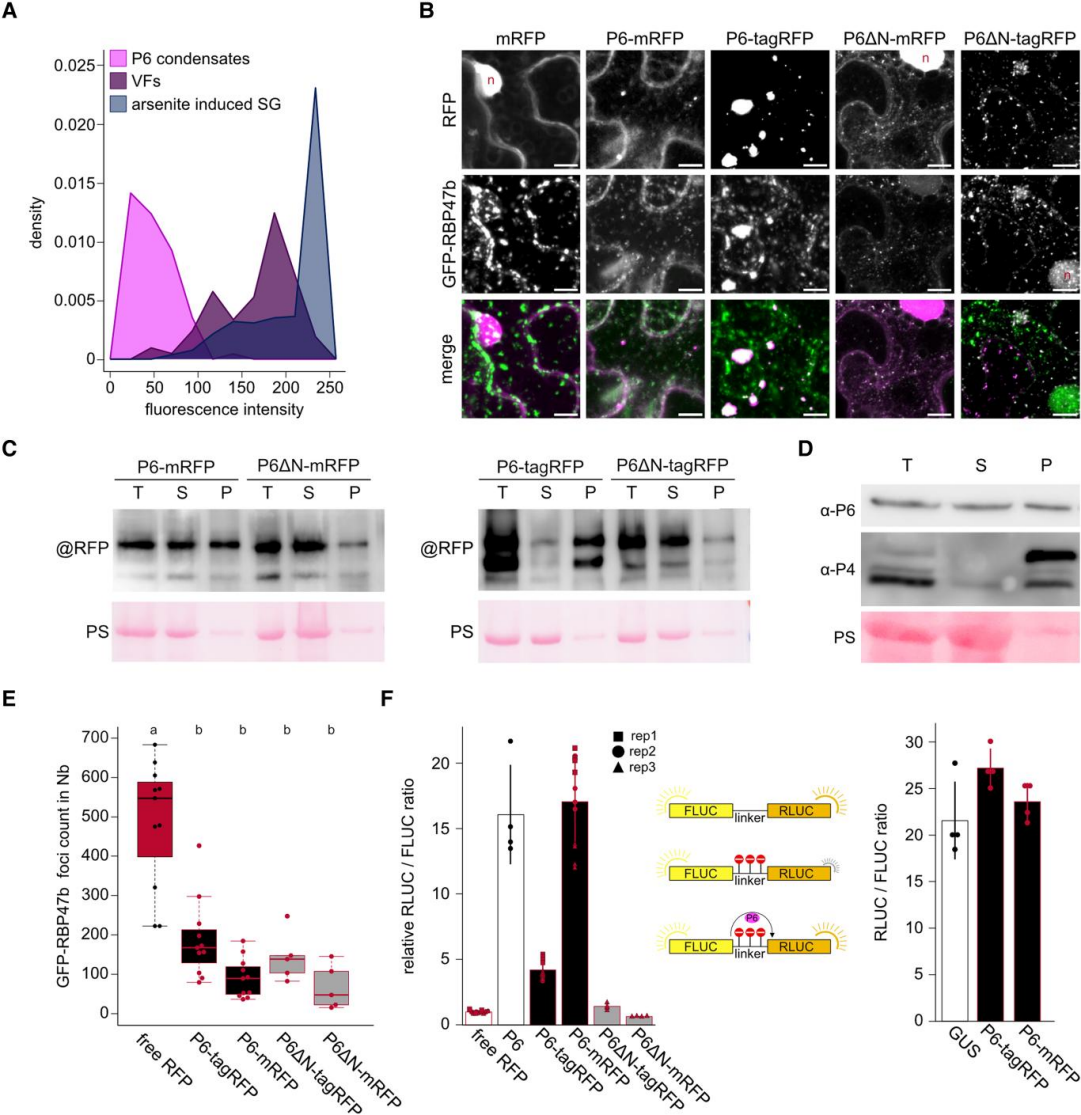
SG inhibition and trans-activation can be uncoupled and are reduced by P6 condensation. **A)** Density polygon (bin width = 25) of GFP-RBP47b fluorescence intensities within preassembled P6-mRFP condensates (*n* = 90) in the transgenic lines (Fig. 7A), VFs from plants at 21 dpi (*n* = 83) and SGs induced using 1 mM arsenite treatment (*n* = 634). **B)** Representative image composed of confocal Z-stack projection of GFP-RBP47b/P6 coexpression in *N. benthamiana* at 3 d after infiltration. The specific P6 constructs are indicated above the images (scale bars = 10 *µ*m). **C)** Immunoblot analysis of the P6 constructs expressed in *N. benthamiana* at 3 dai (days after infiltration). Total (T) protein samples were extracted and subjected to differential centrifugation, resulting in soluble (S) and pellet (P) fraction. Blots were probed with mRFP and tagRFP specific antibodies, respectively. PS staining served as a loading control. **D)** Immunoblot of CaMV proteins P6 and P4 in systemically infected Arabidopsis leaves at 21 dpi probed with specific antibodies. Total (T) protein samples were extracted and subjected to differential centrifugation, resulting in soluble (S) and pellet (P) fraction. PS staining served as a loading control. **E)** GFP-RBP47b foci counts in 100 × 100 *µ*m^2^ regions of *N. benthamiana* (Nb) leaves coexpressing the indicated P6 constructs at 3 dai after a 30 min 38 °C HS treatment. Counts were averaged from 10 (full length constructs) or 5 (P6ΔN constructs) replicates and analyzed with a custom ImageJ pipeline. The box represents the IQR, the solid lines represent the median. Whiskers extend to a maximum of 1.5× IQR beyond the box. **F)** Analysis of transactivation activity for the indicated P6 constructs compared to free mRFP in *N. benthamiana*. P6s and control were coinfiltrated with FLUC-3Xstop-RLUC and luciferase activity was analyzed at 3 dai (left panel). Increased activity of transactivation is indicated by a higher RLUC to FLUC ratio. P6 has no influence on the ratio of RLUC/FLUC when coexpressed with FLUC-linker-RLUC without the stop codons (right panel; *n* = 4). Bars depict mean of values, error bars denote ±Sd. Symbols indicate replicates within 3 independent repetitions.

Some animal viruses sequester SG components to dampen host responses (Emara and Brinton 2007; Panas et al. 2012) and analogously, targeting of SG components to VFs could negatively affect SG formation in a similar manner. However, because the fluorescence intensity of GFP-RBP47b was much lower in the P6-mRFP mock condensates than in VFs (Fig. 8A), this appeared to be the opposite of SG inhibition (Fig. 7A vs. Fig. 2D). A particular feature of P6-mRFP is reduced self-condensation and increased solubility compared to P6-tagRFP, which showed much larger self-condensates and no soluble signal (Fig. 8B). Fractionation by differential centrifugation further supported their difference in solubility (Fig. 8C), and the soluble-to-condensate ratio of P6-mRFP was close to that observed in infected tissue (Fig. 8D). We used this difference in solubility to address how P6 condensation and associated SG component sequestration contribute to inhibited SG formation. We coexpressed P6-mRFP and P6-tagRFP with GFP-RBP47b in *Nicotiana benthamiana* and quantified heat-induced SGs. While the presence of both P6s reduced the amount of SGs, this effect always appeared stronger for the more soluble P6-mRFP (Fig. 8E).

To provide further evidence that the soluble P6 pool is stronger in suppressing SGs than the condensate pool, we introduced a 17 amino acid deletion in the N-terminus region of P6 known to be essential for condensation (P6Ndel3-20, hereafter referred to as P6ΔN) (Haas et al. 2008; Laird et al. 2013) and fused this P6 mutant to both tagRFP and mRFP. As expected, the solubility clearly increased for both fusion proteins compared to the wild type (Fig. 8, B and C). The P6ΔN mutant retained the capacity to suppress heat-induced SG formation when coexpressed with GFP-RBP47b in *N. benthamiana*, and the number of GFP-RBP47b foci was similar to that of the wild-type protein (Fig. 8E).

As we initially hypothesized that P6 may suppress SG formation via both translation-dependent and -independent mechanisms, we assessed the essential activity of P6 and mutants to transactivate the translation of consecutive open reading frames separated by stop codons in the translational reporter construct FLUC-3xSTOP-RLUC (Fig. 8F). P6 coexpression did not alter the FLUC to RLUC ratios when expressed with the control FLUC-linker-RLUC plasmid in *N. benthamiana* (Fig. 8F). P6-mRFP was a much stronger translational transactivator than P6-tagRFP and was comparable with nonfused P6, while the P6ΔN mutants were incapable of transactivation regardless of the RFP tag. These results reveal the importance of the N-terminus of P6 for in planta transactivation. Even more importantly, P6ΔN retained the capacity to suppress SG formation but not for transactivation. This at least partially uncouples these mechanisms to support a translation-independent function of P6 in suppressing SG formation, which we anticipated from the suppression also observed in response to heat stress with full polysome disassembly (Fig. 7). However, these results do not rule out a translation-dependent mechanism that very likely contributes to the suppression of SG formation by altering the stability of polysomes during arsenic stress in P6-mRFP transgenic lines (Fig. 7). Because condensation was reduced in the presence of P6ΔN but SG formation was not suppressed, we propose that soluble P6 suppresses SG formation and that the condensation of P6 is likely to reduce this function, as seen in P6-tagRFP.

## Discussion

Membrane-free condensation of biomolecules into larger entities is a ubiquitous event, including many distinct higher-order complexes of RNA and proteins. Even though this phenomenon has long been recognized, our understanding of the purpose of the actual condensation process has remained largely hypothetical. In the current work, we focused on the formation of specific condensate of CaMV VFs orchestrated by the multifunctional P6 protein. Early work established that VFs lack enclosing membranes and are mainly composed of RNA and proteins with viral particles dispersed within (Martelli and Castellano 1971). VFs were long believed to not exchange much content with their surroundings (Kitajima et al. 1969; Conti et al. 1972), a view that was contradicted by the intriguing finding that viral particles are mobilized from within VFs under conditions mimicking aphid infestation (Bak et al. 2013). Considering that CaMV particles are 50 nm in diameter, VFs must comprise highly flexible matrices in order for them to mobilize, despite our finding that P6 itself is largely static. On the other hand, SG and PB proteins showed rapid mobilities both within and between VFs and their surroundings. This observation supports the notion that VFs are dynamic structures, with G3BP7 and RBP47b showing comparable rates of mobility to their mammalian homologs in arsenite-induced LLPS SGs (Buchan and Parker 2009). Intriguingly, these dynamics change when VFs are subjected to heat but not arsenic stress, including prominent recruitment of the heat-specific SG component eIF4A, a phenomenon widely observed in LLPS organelles (Moore et al. 2011).

VFs grow in size and decrease in number over time, suggesting growth by fusion, a common behavior of phase-separated condensates (Alberti et al. 2019). Furthermore, P6-GFP condensates formed by transient expression in *N. benthamiana* partially responded to the LLPS disruptive agent 1,6-hexanediol (Alers-Velazquez et al. 2021). Interestingly, there appears to be a wide range of variation among LLPS condensates in terms of morphology and properties (Fare et al. 2021); for example, mammalian SGs are frequently irregular in shape and contain substructures (Souquere et al. 2009). The amorphous shape and presence of lacunae suggest that VFs also contain substructures, which vary to some extent between hosts and viral strains (Schoelz and Leisner 2017; Hoffmann et al. 2022). As we determined that VFs contain several SG and PB components, they appear to resemble more general melting pots for RNA metabolic proteins rather than these otherwise canonically distinct LLPS condensates.

**Figure 9.**
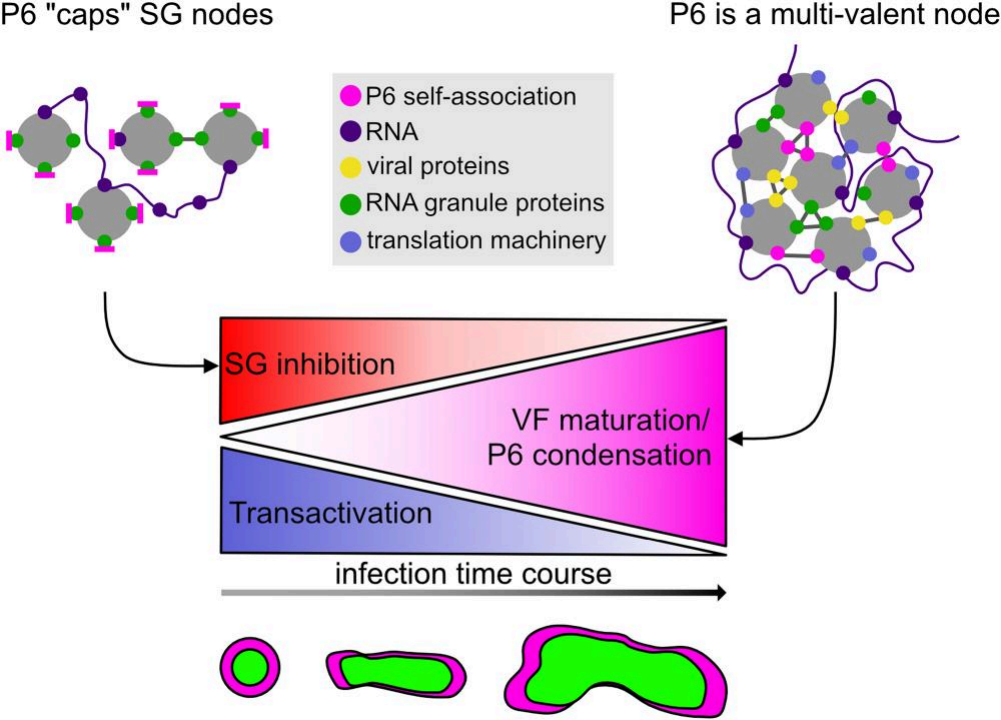
Proposed model for P6 cap and node properties in SGs and VF condensation. The presence of P6 within SGs disrupts the interaction network of canonical SG proteins. P6 occupies the binding sites needed for condensation, thereby acting as a “valence cap” and thus inhibiting the establishment of SGs. On the other hand, SG proteins are sequestered into VFs, where P6 is a multivalent protein that interacts with RNA, viral proteins (including itself), as well as a plethora of host proteins. As such, P6 provides a basis for several interactions, building a matrix that grows over time into mature VFs. The more P6 that is bound within this tight network, the less interaction capacity it has for SG inhibition and translational transactivation, implying a gradual shift in P6 functions during the infection time-course.

The composition of LLPS compartments could be largely controlled by central scaffolding protein nodes that form multivalent interaction networks (Sanders et al. 2020; Fare et al. 2021). Notably, P6 is a truly multivalent node regarding several LLPS criteria: (i) it contains 3 described RNA binding domains; (ii) it shows complex self-association involving at least 4 distinct domains; and (iii) it binds directly to a multitude of proteins including eIF3g and VCS, which we found in VFs (Park et al. 2001; Schoelz and Leisner 2017; Lukhovitskaya and Ryabova 2019; Hoffmann et al. 2022). Scaffolding protein nodes would have reduced mobility compared to recruited clients due to their multiple interactions in the network (Fare et al. 2021), as we observed here for P6 in relation to all other assessed VF components (Fig. 9). Analogously, TSN2 (TUDOR-SN PROTEIN2), which serves as a docking scaffold for SG assembly in plants, also displayed slow mobility in condensates compared to, e.g. RBP47b (Gutierrez-Beltran et al. 2015, 2021). As part of its essential role in CaMV translation, P6 interacts with proteins including ribosomal proteins L13, L18, and L24 (Leh et al. 2000; Park et al. 2001; Bureau et al. 2004) and regulators eIF3g (Park et al. 2001) and TOR (Schepetilnikov et al. 2011). The observation that VFs are coated with ribosomes (Shepherd 1976), together with our finding that eIF3g is extensively localized to VFs, suggest that VFs might function as reservoirs of CaMV translation-supporting factors. Many observations support the possibility that LLPS participates in the formation of VF condensates and that overall, these condensates provide a highly dynamic environment for viral and host factors.

RNA granule proteins within VFs were not responsive to CHX treatment, suggesting that their localization here does not depend on the polysomal release of mRNAs, as do canonical granules (Teixeira et al. 2005; Weber et al. 2008). We found that SG components bound to the extremely abundant nontranslated *8S* viral RNA. The proposed function of *8S* RNA is to serve as a decoy of the plant RNA silencing defense mechanism via the massive generation of small interfering RNAs that are ineffective against the genomic *35S* RNA needed for CaMV replication (Blevins et al. 2011; Hohn 2015). Because *8S* and *35S* RNAs showed comparable binding to RBP47b in vitro, we believe that it is the nontranslatability and shear abundance rather than sequence specificity of *8S* RNA that drives its association with SG components, which is similar to the storage of nontranslating RNA in mammalian cells (Khong et al. 2017). Analogous to serving as decoys of the silencing machinery, we speculate that *8S* binding may sequester SG components from their usual RNA clients and thereby suppress their canonical functions. PB components also localize to the VF matrix, but they have access to the genomic viral *35S* RNA, where they support CaMV translation (Hoffmann et al. 2022).

In multiple instances, animal viruses are able to counteract SG assembly (Lloyd 2016; Poblete-Duran et al. 2016), and some plant viral proteins have also been found to interact and possibly interfere with core SG components (Krapp et al. 2017; Makinen et al. 2017; Reuper et al. 2021). We found suppressed assembly of SGs (monitored via G3BP7 and RBP47b) during heat and arsenic stress in plants with ectopic expression of P6 and that the residual SGs that formed frequently contained P6. We consider 2 nonexclusive modes of inhibited SG formation by P6: translation dependent and translation independent. The translation-dependent mode involves the global stabilization of polysomes to negatively affect SG numbers, as their formation depends on nontranslating RNAs. In support of this idea, the P6Y305P mutant, which is incapable of eIF3g interaction and translation transactivation, did not suppress SG formation or localize to SGs. However, this finding should be carefully considered due to its comparably low accumulation levels. The translation-independent mechanism is supported by the finding that most transgenic P6 lines still showed strongly suppressed SG formation despite the complete polysome disassembly during heat stress. In addition, the P6ΔN mutant was capable of suppressing SG formation but not the transactivation of translation.

The mechanisms by which viral proteins suppress SG formation in mammals are diverse (Lloyd 2016; Poblete-Duran et al. 2016), but the functional analogy between P6 and virus-induced human Adenosine deaminase acting on RNA 1 (ADAR1) is striking. ADAR1 is believed to suppress SG formation via both translation-dependent and -independent mechanisms. ADAR1 localizes to SGs, and its double-stranded RNA-binding domain is essential for translation-independent inhibition of SG formation (Corbet et al. 2021). The double-stranded RNA domain of P6 is known to bind to and activate TOR kinase for translation transactivation and to inhibit autophagy (Schepetilnikov et al. 2011; Zvereva et al. 2016). Interestingly, TOR signaling can promote SG formation in animal models (Sfakianos et al. 2018), highlighting its role as a potential hub to influence SG formation in both a translation-dependent and -independent manner upon P6 manipulation. Further dissection of multifunctional P6 could provide valuable insights into the underlying mechanisms of inhibited SG formation in plants.

Despite the strong inhibition of SG formation by ectopic P6, CaMV-infected tissue still assembles abundant SG-like foci in response to heat and arsenic stress. Evidently, their true SG nature and relatedness to VFs remain unclear due to the frequent presence of P6, suggesting differential P6 regulation during infection. Building on the seminal work of Sanders et al. (2020), we propose that the soluble fractions of P6 acts as a “valence cap,” disrupting canonical interactions and leading to the suppression of SG formation (Fig. 9). By using different fusion proteins of P6 that influence its solubility, we found that the more soluble P6 version was stronger in terms of both transactivation and SG suppression. As there should be a gradual decrease in the amount of soluble P6 via VF condensation during the maturation of an infection in a cell, we propose that the suppression of SG formation and transactivation may decrease and eventually become inactivated through condensation. As P6 is a major determinant of disease symptoms, its condensation may also be important according to the idea of self-attenuation (Schoelz and Leisner 2017), where some plant viruses have been found to limit long-term damage by inactivating their virulence factors (Torres-Barcelo et al. 2008; Zhang et al. 2017; Shukla et al. 2021). For endogenous plant proteins, such a dependency was found for auxin response factor condensation in reducing auxin responsiveness (Powers et al. 2019).

## Materials and methods

### Plant materials and growth conditions

*Arabidopsis thaliana* and *N. benthamiana* plants were grown in walk-in chambers under standard long-day conditions (120 mE, 16 h light/8 h dark cycle) at 22 °C day temperature (20 °C night temperature) and 65% relative humidity for crossing, propagation, and transient expression assays. For infection experiments, plants were grown under short-day conditions (120 mE, 10 h light/14 h dark cycle) at 22 °C day temperature (19 °C night temperature) and 65% relative humidity. Light spectra in both conditions ranged from 400 to 720 nm. All T-DNA and marker lines used in this study were in the Arabidopsis accession Columbia (Col-0) background, which was used as a control for all experiments (Supplemental Tables S1 and S2).

### Plasmid construction, generation of transgenic lines and transient expression

Clones containing *UBP1b*, *UBP1c*, *RBP47b*, *RBP47c*, *RBP45c*, *G3BP7*, and *eIF3g* were PCR amplified from Arabidopsis Col-0 cDNA (Supplemental Table S3) and inserted into the pENTR/D-TOPO cloning plasmid. Entry clones of P6 coding sequences were described before (Hafren et al. 2017) and used for site-directed mutagenesis to obtain Y305P and P6ΔN mutants. The pENTRY clones were recombined into pGWB vectors to generate GFP, mRFP, and tagRFP fusion proteins as well as expression without fusion (Nakagawa et al. 2007) and pUBN/pUBC-DEST for GFP fusion proteins (Grefen et al. 2010). Transgenic Arabidopsis lines were generated by the floral dip method (Clough and Bent 1998); all lines and constructs are listed in Supplemental Table S1. The FLUC-RLUC fusion proteins were generated by constructing a pENTRY/D-TOPO clone containing the coding sequence of *RLUC* preceded by a linker with and without 3 stop codons, which was further recombined with pMCD32:FLUC (Ustun et al. 2018) to create FLUC-linker-RLUC and FLUC-3Xstop-RLUC reporters for the transactivation assay. For transient expression, 5-wk-old grown in long-day conditions *N. benthamiana* leaves were infiltrated with resuspended Agrobacteria (OD 0.2, 10 mM MgCl_2_, 10 mM MES pH 5.6, 150 *µ*M acetosyringone) and the constructs analyzed after 72 h.

### Virus inoculation and quantification

CaMV infection and virus quantification were performed as described by Hoffmann et al. (2022). Viral DNA levels were determined by RT-qPCR and normalized to *18S* ribosomal DNA. RT-qPCR analysis was performed with Maxima SYBR Green/Fluorescein RT-qPCR Master Mix (Thermo Fisher Scientific) using the CFX Connect Real-Time PCR detection system (Bio-Rad) with gene-specific primers (Supplemental Table S4).

### Ribosomal profiling

Transgenic Arabidopsis plants expressing GFP-RBP47b with and without additional expression of P6-mRFP fusion proteins from CaMV strains CM1841 (CMI) and Cabb-BJI (BJI) were vacuum-infiltrated with 1 mM sodium arsenite and incubated at room temperature (RT) for 2 h, heat shocked for 30 min at 38 °C, or left untreated as a control, followed by harvesting in liquid N and storage at −80 °C until processing. Polysome extraction and profile analysis were performed exactly as described in Hoffmann et al. (2022).

### Pull-down assays

Pull-down of GFP-fusion proteins from plants was carried out by grinding 1 g of leaf tissue in 2 mL of buffer (100 mM Tris pH8, 150 mM NaCl, 20 mM KCl, 2 mM MgCl_2_, 1% TX-100, 40 U Ribolock mL^−2^, and protease inhibitor cocktail, Roche) followed by clearing at 17,000 × *g* for 10 min at +4 °C. For FA cross-linking, the tissue was vacuum infiltrated with 1% FA in PBS, cross-linked for 10 min, quenched with 0.125 M glycine PBS, and processed as described above. The lysates were rotated with 25 *µ*L anti-GFP magnetic agarose beads (Chromotech) for 60 min +4 °C, washed 5 times with 1 mL buffer, and eluted with Laemmli sample buffer for immunoblot analysis and TRIzol for RNA isolation. For FA cross-linked RNA, input samples and beads were incubated with protease K for 30 min at +50 °C before isolating RNA using TRIzol. Isolated RNA was treated with DNase, purified, and used for cDNA synthesis followed by RT-qPCR. For in vitro pull-down assays, we generated an RBP47b deletion mutant lacking 100 amino acids from the N-terminus that constitutes the prion domain (Weber et al. 2008) to increase solubility. GST-tagged proteins were bound to glutathione Sepharose, washed with IP buffer (100 mM Tris pH 7.5, 150 mM NaCl, 20 mM KCl, 1 mM MgCl_2_, 1 mM EGTA, 0.05% IpegalC630, and 40 U Ribolock mL^−2^) and incubated with total RNA extracted from CaMV infected plants diluted in IP buffer rotating for 30 min largely as in Dember et al. (1996). After washing 6 times with 1 mL of IP buffer, GST-proteins were eluted with 2 mM reduced glutathione, followed by TRIzol-mediated RNA extraction, cDNA synthesis, and RT-qPCR.

### Transactivation assay

The FLUC-linker-RLUC and FLUC-3Xstop-RLUC were coexpressed with the indicated P6s and controls in *N. benthamiana* by agroinfiltration and harvested 3 dai for analysis. Analysis was performed using the Dual-Luciferase Assay (Promega) according to the manufacturer's instructions.

### Protein detection and fractionation

To detect proteins, immunoblot analysis was performed essentially as described (Hoffmann et al. 2022), with antibodies against GFP (Santa Cruz Biotechnology; sc-9996), tagRFP (Agrisera [RF5R]), mRFP (ChromoTek [6G6]), P4 (Champagne et al. 2004), and P6 (Schoelz et al. 1991). Secondary antibodies were conjugated with horseradish peroxidase (GE Healthcare; NA934 and NA931).

For fractionation of P6 between the soluble and condensate fragments, we homogenized leaf tissue in buffer (100 mM Tris pH8, 150 mM NaCl, and 1% TX-100) to obtain the total sample, followed by centrifugation at 17,000 × *g* for 10 min +4 °C to obtain a supernatant with the soluble P6 and a pellet containing the condensate P6. The pellet was reconstituted in the same buffer using a volume corresponding to the original input. After adding Laemmli sample buffer, all samples were used in immunoblot analysis.

### Confocal microscopy and image processing

Micrographs from leaf abaxial epidermal cells were taken under a Zeiss LSM 800 microscope. GFP and RFP signals were detected at 488 nm/490 nm to 552 nm and 561 nm/569 nm to 652 nm, respectively. Covisualization was achieved through sequential scanning mode. In FRAP analysis, imaging sensitivity was set below saturated fluorescence levels, a 3-s prebleach imaging was followed by bleaching GFP at 488 nm and RFP at 561 nm and, fluorescence recovery was recorded over a 60 s time-course by image acquisition every 1 s. Images were processed with ZEN black software (Zeiss) and ImageJ version 1.53 s. For quantification, Z-stacks were Brightness increased, and a “Gaussian Blur” filter (sigma = 1) was applied. A mask was generated through thresholding, and foci were analyzed using the “Analyze Particles” tool with the settings (size = 0.1 to 2.0, circularity = 0.5 to 1.0 for SGs and size = 2.0-inf for VFs). Colocalization analysis for RBP47b with P6 was performed using the Plugin JACoP (Bolte and Cordelières 2006). The results of FRAP analysis were manually processed in ImageJ. Signal intensities in bleached areas were normalized to an unbleached control area at each timepoint to account for general photobleaching. The time-course series were min–max normalized to eliminate differences in bleaching efficiency and plotted as percent of initial signal vs. time.

### Data analysis and statistical methods

Boxplots/violin plots were constructed with R v4.0.2. The box represents the interquartile range (IQR), the solid lines represent the median. Whiskers extend to a maximum of 1.5× IQR beyond the box. Data were tested for normality using the Shapiro–Wilk test. Statistical comparisons of 2 groups were performed by Welch Two Sample *t*-test with R v4.0.2. One-way ANOVA followed by a post hoc Tukey HSD test (α = 0.05) was performed with R v4.0.2 and the R-package “agricolae” (Version 1.3-3; https://cran.rproject.org/web/packages/agricolae/index.html). Test statistics are shown in Supplemental Table S5.

### Accession numbers

Sequence data from this article can be found in the EMBL/GenBank data libraries under the following accession numbers: LSM1a (AT1G19120), DCP5 (AT1G26110), RBP45a (AT5G54900), RBP45b (AT1G11650), RBP45c (AT4G27000), RBP47a (AT1G49600), RBP47b (AT3G19130), RBP47c (AT1G47490), UBP1a (AT1G54080), UBP1b (AT1G17370), UBP1c (AT3G14100), G3BP7 (AT5G48650), eIF4A (AT1G54270), and eIF3g (AT3G11400).

## Supplementary Material

koad101_Supplementary_Data

## Data Availability

All data is available upon request, no large datasets are part of this work.

## Supplemental data

The following materials are available in the online version of this article.

**Supplemental Figure S1.** P6-mRFP mobility in VFs.

**Supplemental Figure S2.** SG proteins localize in VFs.

**Supplemental Figure S3.** Protein mobility in heat induced SGs.

**Supplemental Figure S4.** Coimmunoprecipitation analysis of viral proteins.

**Supplemental Table S1.** Plant material used and generated in this study

**Supplemental Table S2.** DNA oligonucleotides used in this study for genotyping

**Supplemental Table S3.** DNA oligonucleotides used in this study for molecular cloning

**Supplemental Table S4.** DNA oligonucleotides used in this study for expression analysis

**Supplemental Table S5.** ANOVA table
