# Supplementary material for: Cauliflower mosaic virus protein P6 is a multivalent node for RNA granule proteins and interferes with stress granule responses during plant infection
Source: Plant Cell. 2023 Apr 12;35(9):3363–82. doi: 10.1093/plcell/koad101 (PMC10473198; doi:10.1093/plcell/koad101)
Supplement: koad101_Supplementary_Data [file koad101_supplementary_data.zip › tpc.23.00207Supplemental Figures and Tables.pdf]

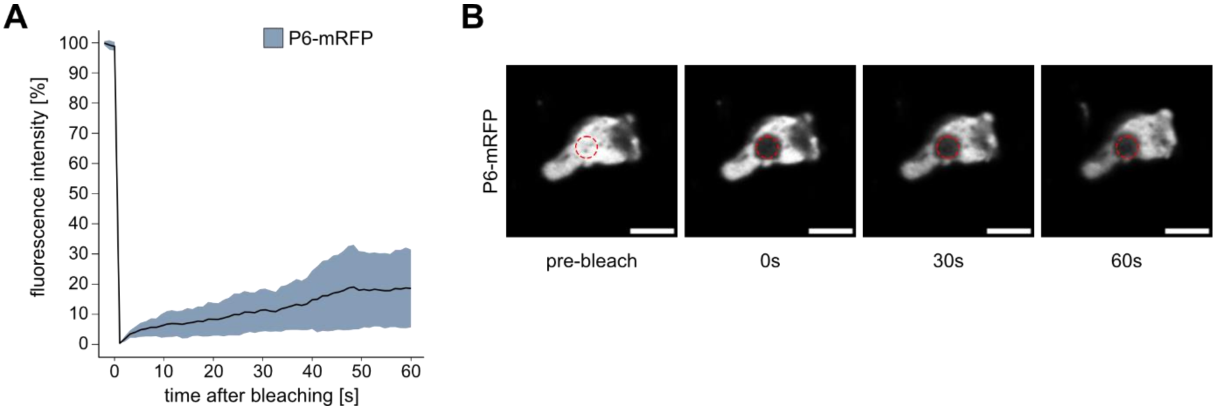

Supplemental Figure S1: P6-mRFP mobility in viral factories. Supports Figure 1. **(A)** FRAP analysis of P6-mRFP (n=14) in viral factories at 21 dpi, showing normalized fluorescence intensities plotted against time after bleaching. Solid lines represent mean, shades denote ± standard deviation. **(B)** Representative images from FRAP analysis in (A) at indicated time-points. Photobleached region is indicated by a red outline. Scale bars = 5 µm.

Supplemental Data. Hoffmann et al. (2023). Plant Cell.

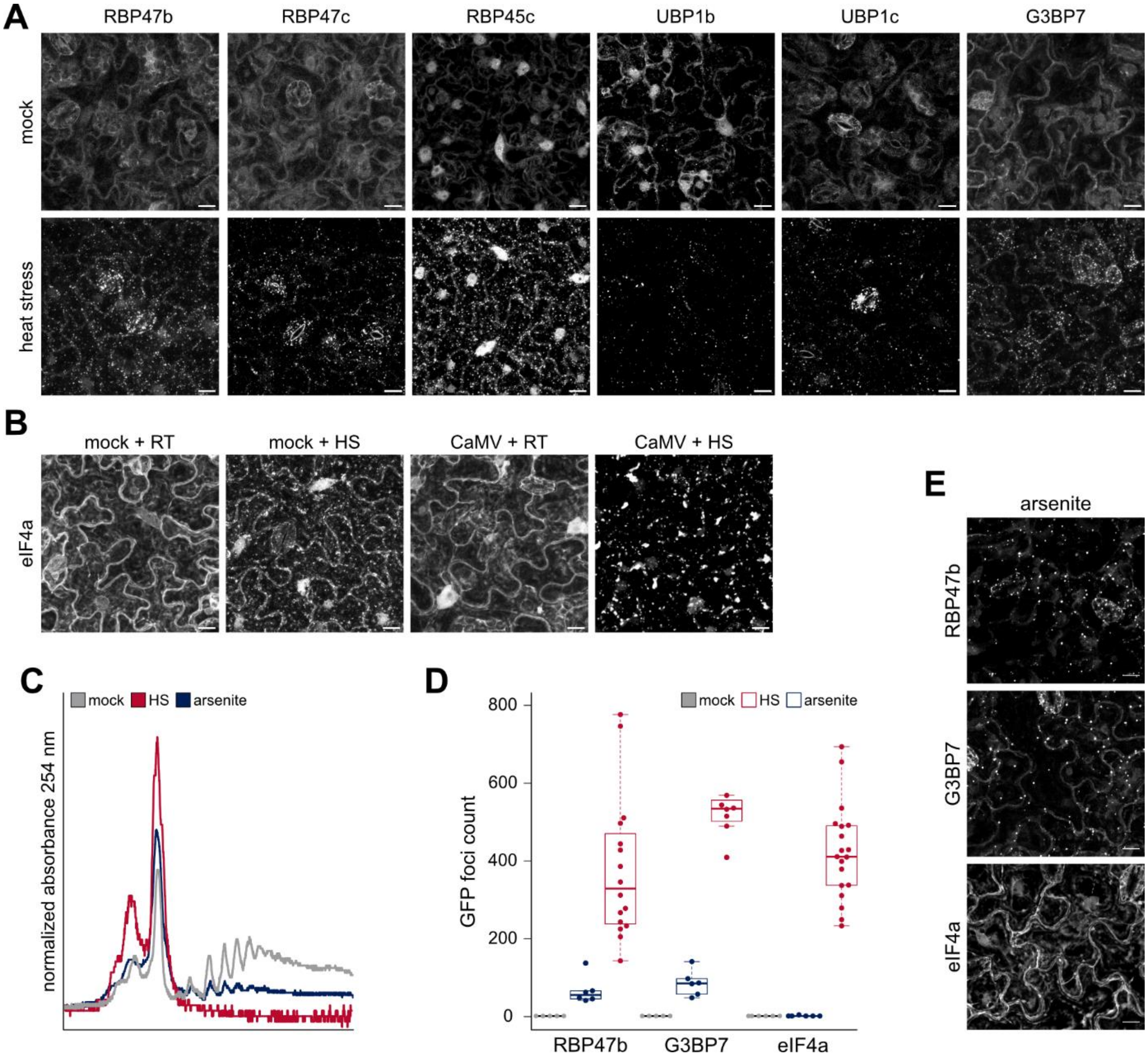

Supplemental Figure S2: Stress granule proteins localize in viral factories. Supports Figure 2. **(A**) Localization of canonical SG markers in uninfected control conditions (upper panel) and after 30 min of 38 °C heat stress (Lower panel). Representative images are composed of confocal z-stack projections (Scale bars = 10 µm).
(B) Localization of eIF4A-GFP in control and heat stress conditions. Representative images are composed of confocal z-stack projections (Scale bars = 10 µm).
(C) Polysome profiles of untreated mock, arsenite or heat stressed GFP-RBP47b plants.
(D) GFP-foci counts before (mock) and after 1 mM 2h arsenite or 30 min of 38 °C heat treatments for GFP-RBP47b, GFP-G3BP7 and eIF4A-GFP in 100 x 100 µm^2. Counts were averaged from at least six replicates with a custom ImageJ pipeline. The box represents the interquartile range (IQR), the solid lines represent the median. Whiskers extend to a maximum of 1.5 × IQR beyond the box.
(B) Localization of SG markers after 1 mM 2h arsenite treatment. Representative images are composed of confocal z-stack projections (Scale bars = 10 µm).

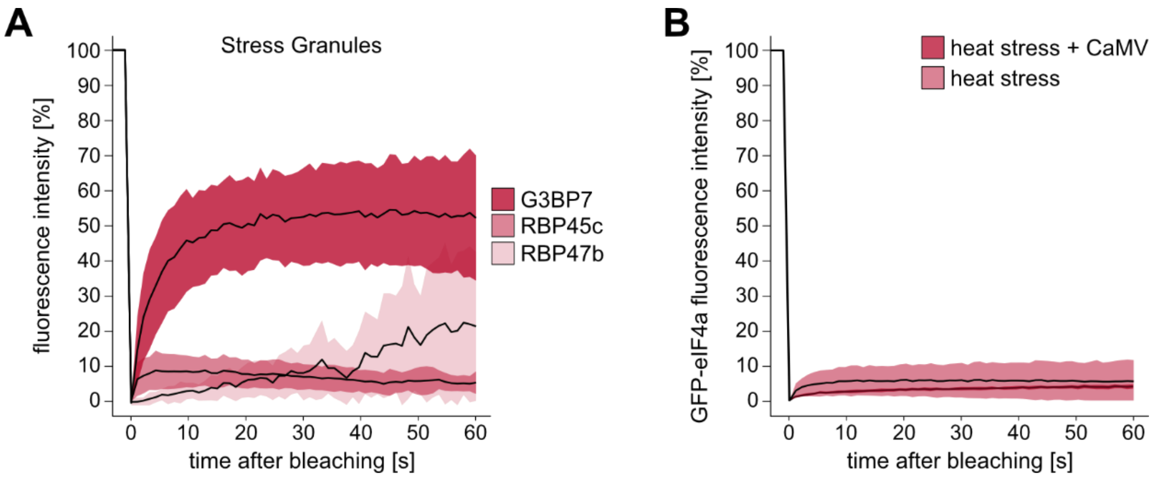

Supplemental Figure S3: Protein mobility in heat induced SGs. Supports Figure 4.
(A) Fluorescence recovery of indicated proteins in SG after 30 min of 38 °C in FRAP analysis of uninfected tissue. Normalized fluorescence intensities are plotted against time after bleaching (n=37-41).
(B) Fluorescence recovery of eIF4A-GFP after photobleaching in VFs (n=7) and SGs (n=60) after 30 min of 38 °C. Normalized fluorescence intensities are plotted against time after bleaching.

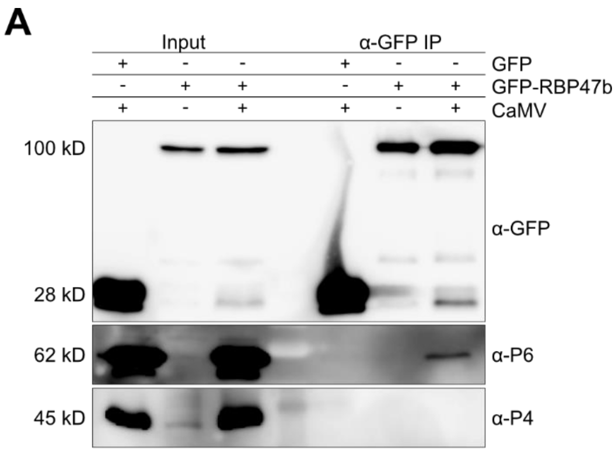

Supplemental Figure S4: Co-immunoprecipitation analysis of viral proteins. Supports Figure 6.
(A) Co-immunoprecipitation analysis of viral proteins P6 and P4 with GFP-RBP47b at 21 dpi with CaMV. Infected plants expressing free GFP and non-infected GFP-RBP47b plants functioned as controls.

Supplemental Table S1. Plant material used and generated in this study

Gene AGI	Gene name	Mutant		Origin
AT5G54900	RBP45A	*rpb45a*	SALK_072359C	NASC
AT1G11650	RBP45B	*rbp45b*	SAIL_505_E03	NASC
AT4G27000	RBP45C	*rbp45c*	SALK_088676C	NASC
AT1G49600	RBP47A	*rbp47a*	SALK_142402C	NASC
AT3G19130	RBP47B	*rbp47b*	GABI_626C01	NASC
AT1G54080	UBP1A	*ubp1a*	GK-625F11	Obtained from Julia Bailey-Serres
AT1G17370	UBP1B	*ubp1b*	GABI_262E01	Obtained from Claudia Köhler
AT3G14100	UBP1C	*ubp1c-1*	SAIL_363_G02	Obtained from Julia Bailey-Serres
**Gene AGI**	**Line name**	**Vector used**	**Background**	**Origin**
AT1G26110	DCP5-GFP			Chicois et al., 2018
AT1G19120	LSM1a-GFP			Hoffmann et al., 2022
AT4G27000	GFP-RBP45c	pUBN-GFP	Col-0	this study
AT3G19130	GFP-RBP47b	pUBN-GFP	Col-0	this study
AT1G47490	GFP-RBP47c	pUBN-GFP	Col-0	this study
AT1G17370	GFP-UBP1b	pUBN-GFP	Col-0	this study
AT3G14100	GFP-UBP1c	pUBN-GFP	Col-0	this study
AT5G48650	GFP-G3BP7	pGWB506	Col-0	this study
AT3G11400	eIF3g-GFP	pUBC-GFP	Col-0	this study
AT1G54270	eIF4A-GFP			Hamada et al., 2018
CaMV P6	47bP6	pGWB554	GFP-RBP47b	this study
CaMV P6	G3P6	pGWB654	GFP-G3BP7	this study
CaMV P6	47bP6T305P	pGWB554	GFP-RBP47b	this study

Supplemental Data. Hoffmann et al. (2023). Plant Cell.

Supplemental Table S2. DNA oligonucleotides used in this study for genotyping

Gene AGI	Mutant		Name	Sequence
-	-	-	SALK_LBb1.3	ATTTTGCCGATTTCGGAAC
-	-	-	SAIL_LB	GCCTTTTCAGAAATGGATAAATAGCC TTGCTTCC
-	-	-	GABI_LB_08760	GGGCTACACTGAATTGGTAGCTC
AT5G54900	*rbp45a-1*	SALK_072359C	SALK_072359C_F	TTTGAGCCGTTTGACCAATAG
			SALK_072359C_R	TTTGCAAACGTTGTTTGAGTG
AT1G11650	*rbp45b*	SAIL_505_E03	SAIL_505_E03_F	CGCAAGAAAACGAGCAAGTAG
			SAIL_505_E03_R	ACAAACATTCAACAACGCTCC
AT4G27000	*rbp45c*	SALK_088676C	SALK_088676C_F	TCTCGAAGTTGGATCTTGCAG
			SALK_088676C_R	CCATTGTCAAACGGTTACAGC
AT1G49600	*rbp47a*	SALK_142402C	SALK_142402C_F	TCAAAAGTTGATGTCAACCCC
			SALK_142402C_R	TGGCAATGTACTTTTCTTGCC
AT3G19130	*rbp47b*	GABI_626C01	GABI_626C01_F	AGTTTGGCATCACTGAACCAC
			GABI_626C01_R	CTCTCTCTCTCTCTCCGCCTC
AT1G54080	*ubp1a*	GK-625F11	GK-625F11_F	ACTACCGAAAAGCTTCATTCG
			GK-625F11_R	TATATTAGTTTGGGCTGGGCC
AT1G17370	*ubp1b-3*	GK-926G07-06	GK-926G07-06_F	TTTTTGAGCTTATTGTAATTGGT
			GK-926G07-06_R	CAAGAACACTGTAGGAACAACGA
AT3G14100	*ubp1c*	SAIL_363_G02	SAIL_363_G02_F	AATCAGTCACAAAGACCGGAC
			SAIL_363_G02_R	CAAAACCAGGAGGAAGGTTTC

Supplemental Data. Hoffmann et al. (2023). Plant Cell.

Supplemental Table S3. DNA oligonucleotides used in this study for molecular cloning

Name	Sequence 5'-3'	Application
RLUC F	CACCATGACTTCGAAAGTTTATGAT	amplify RLUC from plasmidpMDC32::RLUC described in Ustun et al., 2018.
RLUC R	TT GTTCATTTTTGAGAACTCGC	
linker Rluc nested F	TGCTGGAGCTGGATCTatgacttcgaaagtttat gat	amplify from RLUC plasmid with 512, adds first part of linker for later nested PCR
STP3linker Rluc nested2 F	caccTAGTAGTAGGCCTCAGCCGGTGCTGGAGCT GGATCT	adds 3x stop codons (TAG) first part of linker after nested PCR amplify with RLUC R
UBP1b F	CACCATGCAGAGGT TGAAGCAGCA G	pENTRY/D-TOPO cloning of At
UBP1b R	TTACTGGTAGTACATGAGCTG	pENTRY/D-TOPO cloning of At
UBP1c F	CACCATGCAGAATC CGAGACTGAA G	pENTRY/D-TOPO cloning of At
UBP1c R	TTACTGATAGTACATGAGTTGCTG	pENTRY/D-TOPO cloning of At
Rbp47b F	CACCATGCAGACAACCAACGGCTCA	pENTRY/D-TOPO cloning of At
Rbp47b R	TGGGTGGATTCTCCCCATGATAGTTGTTG	pENTRY/D-TOPO cloning of At
Rbp47c F	CACCATGGCAGACG TCAAGATTCA ATC	pENTRY/D-TOPO cloning of At
Rbp47c R	TCAGCTAACTTGTTGCTGATGAC	pENTRY/D-TOPO cloning of At
Rbp45c F	CACCATGATGCAGCAGCCACCTCCAGCT	pENTRY/D-TOPO cloning of At
Rbp45c R	TCACTGCTGTTGCTGCTGGTATCC	pENTRY/D-TOPO cloning of At
eIF3G1_F	CACCATGACGATCG ATTCGCAGCA	pENTRY/D-TOPO cloning of At
eIF3G1_R	GGTTGGTCTTGGAGTTG	pENTRY/D-TOPO cloning of At
G3BP7 F	TTTTGCGGCCGCCATGATGGCGACTCCTTATCCT GG	pENTRY cloning of At using NotI and SgsI insertion
G3BP7 R	TTTTGGCGCGCCCGCGACCACCACCGCGGTAGTA ACC	pENTRY cloning of At using NotI and SgsI insertion
P6 TYR305Pro_F	CCCCCGAGTAACAATCTCCAGGA	site-directed mutagenesis by outward PCR on pENTRY/D-TOPO P6 B-JI clone
P6 TYR305Pro_R	GATCGTCTTGATGAGACCTG	site-directed mutagenesis by outward PCR on pENTRY/D-TOPO P6 B-JI clone
P6 deltaN3-20 F	CACCATGGAGAACAAAATAAGCTTAGCAAG	pENTRY/D-TOPO cloning of At
P6 deltaN3-20 R	ATCCACTTGCTTTGAAGACGT	pENTRY/D-TOPO cloning of At

Supplemental Table S4. DNA oligonucleotides used in this study for expression analysis

Name	Sequence 5'-3'	Application
PP2A F	TAACGTGGCCAAAATGATGC	house keeping gene
PP2A R	GTTCTCCACAACCGCTTGGT	house keeping gene
Genomic 18S rDNA F	CGTGATCGATGAATGCTACC	house keeping gene
Genomic 18S rDNA R	GGGGTTTGTTGCACGTATTA	house keeping gene
CaMV DNA F	AGCGGTCAAAATATTGCTTA	virus accumulation
CaMV DNA R	AACTTACCGTATGCTAGATTACCT	virus accumulation
RTqPCR 8S F	TCACCAGTCTCTCTCTACAAATCTATCTC	virus accumulation
RTqPCR 8S R	GCGAAACCCTATAAGAACCCTAATTCC	virus accumulation
RTqPCR 35S F	GGTGAAGGACCATCTAGATAC	virus accumulation
RTqPCR 35S R	CAATCCGATTTCTGCTGCCCA	virus accumulation
18s rRNA F	CTAGAGCTAATACGTGCAACAAAC	RIPA normalization
18s rRNA R	GAATCGAACCCTAATTCTCCG	RIPA normalization

Supplemental Table S5. ANOVA Table

		Df	Sum Sq	Mean Sq	F-value	P-value	
**Figure 1B**		Df	Sum Sq	Mean Sq	F-value	P-value	
	Treatment	5	337008	67402	31.66	3.14e-12	***
	Residuals	36	76631	2129			
**Figure 1C**		Df	Sum Sq	Mean Sq	F-value	P-value	
	Treatment	5	110.40	22.08	76.27	<2e-16	***
	Residuals	36	10.42	0.29			
**Figure 2D**		Df	Sum Sq	Mean Sq	F-value	P-value	
	Treatment	7	1900127	271447	90.5	<2e-16	***
	Residuals	64	191955	2999			
**Figure 5A**		Df	Sum Sq	Mean Sq	F-value	P-value	
	Treatment	9	1.399	0.15544	3.405	0.000929	***
	Residuals	120	5.478	0.04565			
**Figure 5A**		Df	Sum Sq	Mean Sq	F-value	P-value	
	Treatment	5	2.126	0.4253	11.34	6.9e-08	***
	Residuals	65	2.437	0.0375			
**Figure 7A (left panel)**		Df	Sum Sq	Mean Sq	F-value	P-value	
	Treatment	4	649306	162326	15.72	4.03e-08	***
	Residuals	45	464680	10326			
**Figure 7A (right panel)**		Df	Sum Sq	Mean Sq	F-value	P-value	
	Treatment	4	1540027	385007	14.52	9.65e-08	***
	Residuals	46	1219648	26514			
**Figure 7E**		Df	Sum Sq	Mean Sq	F-value	P-value	
	Treatment	3	77324	25775	7.428	0.00156	**
	Residuals	20	69398	3470			
**Figure 8E**		Df	Sum Sq	Mean Sq	F-value	P-value	
	Treatment	4	1113764	278441	25.37	2.75e-10	***
	Residuals	38	416984	10973			
