# Supplementary material for: Cauliflower mosaic virus protein P6 is a multivalent node for RNA granule proteins and interferes with stress granule responses during plant infection
Source: Plant Cell. 2023 Apr 12;35(9):3363–82. doi: 10.1093/plcell/koad101 (PMC10473198; doi:10.1093/plcell/koad101)
Supplement: koad101_Supplementary_Data [file tpc23.00207.PeerReviewReport.pdf › tpc23.00207.PeerReviewReport.pdf]

Cauliflower mosaic virus protein P6 is a multivalent node for RNA granule proteins and interferes with stress granule responses during plant infection

Gesa Hoffmann, Silvia López-González, Amir Mahboubi, Johannes Hanson, and Anders Hafrén

Corresponding author: Anders Hafrén, anders.hafren@slu.se

Review timeline:

TPC2022-RA-01032	Submission received:	Oct. 21, 2022
	1st Decision:	Nov. 23, 2022 *manuscript declined*
**TPC2023-RA-00207**	Submission received:	Mar. 2, 2023
	1st Decision:	Mar. 22, 2023 *acceptance pending, sent to science editor*
	Final acceptance:	April 4, 2023

REPORT: (The report shows the major requests for revision and author responses. Minor comments for revision and miscellaneous correspondence are not included. The original format may not be reflected in this compilation, but the reviewer comments and author responses are not edited, except to correct minor typographical or spelling errors that could be a source of ambiguity.)

TPC2022-RA-01032 1st Editorial decision – *declined* **Nov. 23, 2022**

Thank you for choosing to send your manuscript entitled "Cauliflower mosaic virus protein P6 forms a microenvironment for RNA granule proteins and interferes with stress granule responses" for consideration at The Plant Cell. Your submission has been evaluated by members of the editorial board as well as expert reviewers in your field, and we regret to inform you that we are not able to recommend the publication of this manuscript in its current form. We have had input from multiple scientists and we have solicited postreview comments. That said, we would be willing to re-consider a new manuscript that fully addressed the concerns raised during this review process. If you decide to resubmit to The Plant Cell, it will be evaluated as a new submission subject to full assessment by the editorial board, and if sent for external review, a new set of reviewers might be chosen. Reviewers will be asked to assess your work as a new manuscript (i.e. are the claims fully supported by the data and do the results presented move the field forward?), and not only whether previous reviewer comments have been addressed. Although Reviewer #2 was very enthusiastic, Reviewer #1 felt that the work did not meet the level of impact typical of a Plant Cell paper. In particular, they felt the work presented is unable to show that the interaction between these new SG proteins and P6 has any effect on CaMV infection, or that P6 modulates SG responses in a manner that affects the capacity of the plant to survive stress conditions. I encourage you to consider experiments that address this concern, even if it was not presented in the critique text below.

-- Reviewer comments:

[Reviewer comments shown below along with author responses]

TPC2023-RA-00207 Submission received **Mar. 2, 2023**

Reviewer comments on previously declined manuscript and **author responses:**

Reviewer #1:

In this manuscript the authors have examined the potential interactions between the CaMV virion factories that form from aggregation of the P6 protein and host stress granules. The authors find that several stress granule proteins are incorporated into the P6 virion factories, and that some P6 protein ends up in stress granules. They also find that P6 reduces the assembly of stress granules, and in particular, this is more pronounced with P6-RFP, a fusion protein between P6 and RFP that has a more soluble form than P6-GFP. The authors also found the more soluble version of

P6 supported a higher level of transactivation and was a stronger suppressor of SG formation than the form of P6 that was more prone to condensation.

The authors tested the capacity of CaMV to infect T-DNA knockouts of SG components in Arabidopsis, finding that CaMV accumulation was unaffected by any of the gene knockouts. CaMV DNA levels were unaffected even in a triple knockout. Similarly, although P6 could suppress SGs and stabilize polysomes, these effects could not be linked to any evident phenotype associated with stress granules. The authors note that SGs influence mRNA translation, storage and decay, and also serve as protective refuges during stress. Although CaMV interacts with several stress granule proteins, this interaction does not appear to affect the plant's capacity to respond to abiotic stresses such as heat, drought, salt, or biotic stresses associated pathogen infections. By contrast, CaMV P6 has been shown to alter several important plant stress pathways, such as the SA, JA and ethylene.

R1.1: Does the interaction of P6 with plant stress proteins contribute to the regulation of any of the plant stress responses?

> **Our model has been updated to propose how P6 may interact and suppress SGs according to reviewer 2, which we agree to be a plausible expansion to our early interpretation of P6 being a multivalent node that was largely inferred from the different component mobilities in viral factories. However, the outcome of SG suppression/co-option by P6 remains currently unclear and may as we discuss be a process that changes along the maturation of VFs and P6 condensation. This is definitely a future detail we will try to unravel but of course quite complicated according to the multifunctional nature of P6 with so many different cellular interactions as we discuss in the manuscript.**

R1.2: Similarly, the authors show that P6-RFP is more soluble than P6-GFP, and this form has a greater effect in suppressing SGs and in transactivating translation, a core function of P6. However, it is unclear how this relates to the unmodified P6. Are the properties of the unmodified P6 more closely aligned with the version tagged with GFP or RFP? The answer to this question is important for evaluating the model presented in Figure 8.

> **We agree that this is a very important question! We have complemented the data of Figure 8C and 8D, which shows that untagged P6 behaves like P6-mRFP rather than P6-tagRFP with the transactivation assay (Figure 8F). While the transactivation capability of P6-mRFP falls in the same range as untagged P6 and higher than P6-tagRFP, we considered untagged (blind) P6 unsuitable for similar microscopy analysis of SG suppression as performed for the red fusions. Overall, this data supports that P6-mRFP better recapitulates native P6 functions and is in line with its prominent condensation during infection Fig.1. Of note, we have also observed successful complementation of CaMV carrying a defective P6, by both P6-mRFP and P6-tagRFP. However, because we do not know yet what is the exact molecular defect of the P6 mutant CaMV that is being complemented, we do not find this result mature enough to strengthen the current manuscript, but this data can be provided upon request.**

R1.3: P6 has been shown to interact with a number of different plant proteins, and it is interesting that this number now includes stress granule proteins. However, this paper is unable to show that the interaction between these new SG proteins and P6 has any effect on CaMV infection, or that P6 modulates SG responses in a manner that affects the capacity of the plant to survive stress conditions.

> **It is overall very difficult to extrapolate SG specific effects in plants owing to the lack of a general genetic system and multigene family nature of both core families of SG nucleating UBP1 and G3BP7. We do believe that we have provided good evidence to suggest that SGs are counteracted by P6, via both translation-dependent and independent ways. This is not mutually exclusive with a presence of SG factors in VFs and P6 containing “SGs” induced by additional stress to CaMV infected tissue, a view we have tried to clarify including an updated model in Figure 9. Notably, a global translation inhibition as the most “evident” canonical phenotype of SGs is clearly not operating as polysomes are increased in CaMV infected tissue.**
>
> **That said, we fully agree that this was one of the outstanding open questions of the submitted work. While T-DNA mutants did not show any clear effects on virus accumulation, we now tried to answer this request by analyzing phenotypes in a collection of SG-component over-expression lines. We find different degrees of stunting for UBP1 family members, reduced infection rates and a trend towards less virus accumulation. We have incorporated this data and think our initial view that the SG machinery is largely neutralized in CaMV infection is still valid.**

There are a few figures that are mislabeled.

Pg. 7, lines 222-223 and Fig. 4. The narrative refers to Figure 4H, but Figure 4 does not have a panel H.

Pg. 8, lines 256 and 257, and Fig. 5. The narrative refers to Figure 5F-H. I believe the authors intend Figure 5G-I. Also, the narrative refers to Figure 5I. I believe the authors intend Figure 5F.

We apologize for these mistakes and have corrected them in the manuscript.

Reviewer #2:

Hoffmann et al. present an interesting study focused on the interplay between viral factories by the cauliflower mosaic virus and stress granules in Arabidopsis. Viral factories are membraneless assemblies that occur during infection by a multitude of viruses. These biomolecular condensates are intimately tied to viral replication. Viral factories are potentially catalytic by concentrating the viral components to promote reliable packaging of the viral genome. Additionally, they can interfere with innate immune responses and cellular defense systems, such as stress granule assembly (which is known to sequester viral RNAs). The evolutionary arms race between host and pathogen has resulted in the emergence of several pathways by which viruses can interfere with or corrupt such human condensates. In this study, the authors explore the case of the CMV P6 protein. Hoffmann et al. show that CMV viral factories can be reconstituted by expressing P6, arguing for its role as a scaffold, and that these condensates recruit several--but not all--stress granule factors. This observation is in line with results for other viral factories. Additionally, P6 can inhibit canonical stress granule assembly. Other studies have previously observed this for viral proteins that act as competitive G3BP1 inhibitors. For P6, the authors make a case for eIF3g binding as the inhibitory mode of action. In all, the authors present a study that is well-executed and provides an interesting new way of how viral proteins can interfere with stress granule function. If the authors address two minor comments, I can only give my strongest endorsement for immediate publication in The Plant Cell.

We can only express our gratitude and happiness for these warming words.

1. The title could be stronger. "Microenvironment" is vague, undefined, has no biological meaning, and hence detracts from the actual data. Better would be a title that directly summarizes the findings: For example, "Cauliflower mosaic virus protein P6 drives viral factory formation and inhibits stress granule assembly."

We have taken your advice and provide a revised title.

2. The authors would need to acknowledge the landmark work by Sanders et al. (2020, Cell). This study provides a network-based framework on how to think about condensate specificity. Given that P6 can interfere in a translation-independent manner with stress granule formation, it seems to act as a "cap". In regards to its own condensation, it will act as a "node".

We thank the reviewer for pointing us further in this direction, and have incorporated this theory into our hypothetical model. We have not gone as far to propose that the cap activity may actually go via eIF3g based on our P6^{T305P} mutant results, as this is still at least close to impossible to uncouple from the translation transactivation process.

TPC2023-RA-00207 1st Editorial decision –*acceptance pending* **Mar. 22, 2023**

We are pleased to inform you that your paper entitled "Cauliflower mosaic virus protein P6 is a multivalent node for RNA granule proteins and interferes with stress granule responses" has been accepted for publication in The Plant Cell, pending a final editorial review by a science editor.

Final acceptance from Science Editor **April 4, 2023**
